# Reactive Carbide‐Based Synthesis and Microstructure of NASICON Sodium Metal All Solid‐State Electrolyte

**DOI:** 10.1002/adma.202512961

**Published:** 2025-11-05

**Authors:** Callum J. Campbell, Scott Monismith, Vikalp Raj, Yixian Wang, Qianqian Yan, Cole D. Fincher, Rohit Raj, Yet‐Ming Chiang, John Watt, Josefine D. McBrayer, David Mitlin

**Affiliations:** ^1^ Materials Science and Engineering Program & Texas Materials Institute (TMI) The University of Texas at Austin Austin TX 78712 USA; ^2^ Power Sources Technology Group Sandia National Laboratory Albuquerque NM 87185 USA; ^3^ Department of Materials Science & Engineering Massachusetts Institute of Technology Cambridge MA 02139 USA; ^4^ Center for Integrated Nanotechnologies Los Alamos National Laboratory Los Alamos NM 87545 USA

**Keywords:** dendrite growth, microstructure, NASICON, sodium‐metal batteries, solid‐state batteries

## Abstract

Reactive carbide precursor‐based synthesis of NASICON‐type NZSP (Na_1+x_Zr_2_Si_x_P_3‐x_O_12_) solid‐state electrolyte (SSE) is demonstrated, in contrast to the established oxide‐based approach. Exothermic decomposition of ZrC and SiC in air homogenizes microstructure, yielding 98% compact density after conventional sintering at 1200 °C. Quantitative stereology demonstrates that significant microstructural differences are present. Compacts of carbide‐derived Carb‐NZSP are 98% dense with a secondary zirconium oxide (ZrO_2_) volume fraction of 0.2% ± 0.3%, versus 93% dense and 3% ± 1% for oxide‐derived baseline. For Carb‐NZSP, the secondary glassy phosphate phase is agglomerated, while for baseline, it is dispersed and percolated. Electrochemical testing combined with post‐mortem analysis demonstrates how microstructural control of secondary phases is critical for dendrite suppression: Carb‐NZSP critical current density (CCD) is 3.1 ± 0.8 mA cm^−^^2^ at 0.1 mAh cm^−^^2^, versus 1.0 ± 0.7 mA cm^−2^ at 0.1 mAh cm^−2^. Cryogenic focused ion beam (cryo‐FIB) analysis demonstrates that in both materials, the porous 2D sheet‐like sodium metal dendrites propagate around and subsume NZSP grains, likely following a path enriched with glassy phase and with porosity. Dendrites also flow around isolated zirconia particles. Phase field simulation reveals deflection of dendrites by mechanically tough zirconia, while brittle glassy phase accelerates dendrite growth, especially when finely distributed.

## Graphical abstract

Sodium Metal All‐Solid State Batteries (Na‐ASSBs) are enabled by the synthesis of the solid state electrolyte, NASICON (Na_1+x_Zr_2_Si_x_P_3‐x_O_12_), using carbide‐based precursor compounds (ZrC and SiC); resulting in dense, pure, and mechanically improved microstructure. Heat released during solid state synthesis creates a uniform microstructure with a favorable distribution of secondary impurities – enabling faster current capabilities without dendrite driven short circuit.

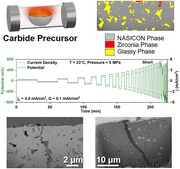

## 1 Introduction

Sodium solid‐state batteries (Na‐SSBs) are an emerging alternative to lithium‐ion batteries that have dominated the energy storage market for the last three decades.^[^ ^1^, ^2^, ^3^, ^4^, ^5^, ^6^ ^]^ Compared to lithium, sodium is abundant and evenly distributed in the Earth's crust, making it an attractive candidate for energy storage solutions, such as sodium‐ion and sodium‐metal batteries.^[^ ^7^ ^]^ However, unlike lithium, sodium cannot intercalate effectively into graphite,^[^ ^7^, ^8^ ^]^ necessitating the development of alternative anodes.^[^ ^9^, ^10^, ^11^ ^]^ Sodium metal stands out with its exceptionally high theoretical energy density (1166 mAh g^−1^). Yet, it faces significant challenges, including high intrinsic reactivity and dendrite growth when used with liquid electrolytes.^[^ ^7^, ^8^ ^]^ While some liquid electrolytes have demonstrated the ability to support uniform sodium deposition during cycling, their high flammability, particularly with ether‐based solvents, remains a major hurdle for commercialization.^[^ ^12^, ^13^ ^]^ Non‐flammable solid‐state electrolytes (SSEs) provide a pathway to safer operation, making them promising candidates for sodium metal anodes.^[^ ^14^, ^15^, ^16^, ^17^, ^18^, ^19^ ^]^

Among the various solid‐state electrolytes, the inorganic oxide Na_1+x_Zr_2_Si_x_P_3‐x_O_12_ (Sodium SuperIonic CONductor, NASICON) has garnered significant attention since its discovery by Hong and Goodenough.^[^ ^20^, ^21^ ^]^ This material, particularly the Na_3_Zr_2_Si_2_PO_12_ (NZSP) stoichiometry, exhibits a highly Na ion conductive monoclinic phase, with room‐temperature ionic conductivity typically ranging between 10^−4^ and 10^−3^ S cm^−1^.^[^ ^22^, ^23^, ^24^, ^25^, ^26^, ^27^, ^28^, ^29^, ^30^, ^31^ ^]^ Such high ionic conductivity positions NZSP as a promising candidate for sodium metal all‐solid‐state batteries. Efforts to improve NZSP have largely focused on bulk doping,^[^ ^32^, ^33^, ^34^, ^35^, ^36^, ^37^, ^38^, ^39^, ^40^, ^41^ ^]^ optimizing sintering conditions,^[^ ^42^, ^43^, ^44^, ^45^ ^]^ and interface modifications.^[^ ^25^, ^27^, ^46^, ^47^, ^48^, ^49^, ^50^, ^51^, ^52^ ^]^ Doping strategies, including aliovalent doping at Zr^4+^ octahedral sites and polyanion substitution in SiO_4_ and PO_4_ tetrahedral sites,^[^ ^33^, ^53^, ^54^, ^55^, ^56^ ^]^ have significantly enhanced ionic conductivity. Conductivities above 1 and up to 5 mS cm^−1^ have been reported when using synthesis methods like sol‐gel in combination with polyanion and aliovalent metal cation doping.^[^ ^55^, ^56^, ^57^, ^58^, ^59^, ^60^ ^]^

Despite these advances, NZSP is persistently plagued by a number of challenges such as a poor interface with sodium metal^[^ ^52^, ^61^, ^62^, ^63^, ^64^ ^]^ and the presence of secondary phases such as zirconia (ZrO_2_) and amorphous glassy phases.^[^ ^53^, ^65^, ^66^ ^]^ The secondary phase impurities that arise due to synthesis challenges are nearly ubiquitous across both traditional solid‐state and solution‐assisted synthesis methods.^[^ ^67^ ^]^ The zirconia phase, in particular, can impede ionic transport, leading to inferior electrochemical performance. It has been suggested that zirconia particle size, morphology, and distribution may be responsible for differences in the mechanical properties of NZSP, especially fracture toughness – a parameter closely associated with dendrite tolerance.^[^ ^68^, ^69^ ^]^ Synthesis methods to reduce the quantity of zirconia have been reported, but largely rely on the production of an intentionally zirconium deficient NASICON.^[^ ^70^ ^]^ Addressing these challenges requires not only reducing the impurity content but also comprehensively evaluating its effect on dendrite suppression and interfacial stability. Recent advancements in solid‐state synthesis aim to mitigate these issues by producing dense, high‐purity NZSP through techniques such as ultrafast joule heating,^[^ ^26^ ^]^ spark plasma sintering^[^ ^43^ ^]^ high‐energy milling.^[^ ^45^ ^]^ However, these methods often involve high processing temperatures that can volatilize sodium and phosphorus, exacerbating the formation of secondary phases.^[^ ^32^, ^48^, ^71^ ^]^ The introduction of sintering aids, including Na_2_SiO_3_, CuO, and Na_3_BO_3_, has been shown to improve compact densification, total ionic conductivity, and critical current densities.^[^ ^22^, ^44^, ^72^ ^]^

In this study, we present the first reported use of reactive carbide precursors, ZrC and SiC, for the solid‐state synthesis of NZSP. This novel approach leverages an exothermic reaction pathway that facilitates improved densification, phase purity, and microstructural distribution. We employ a carbide‐based approach for the synthesis of NASICON‐type NZSP solid‐state electrolyte and demonstrate it against an established oxide precursor method. Quantitative stereology reveals significant differences in volume fraction, morphology, and distribution of secondary zirconia and glassy phosphate phases in Carb‐NZSP versus in baseline NZSP. Electrochemical testing combined with post‐mortem analysis demonstrates how microstructural control of secondary phases is critical for dendrite suppression. The sheet‐like sodium metal dendrites appear to propagate around the NZSP grains. These are the regions that would be enriched in the glassy phase and should possess higher levels of porosity. Phase field simulations of dendrite propagation reveal the deflection of dendrites by zirconia particles; while the presence of brittle phosphate glass accelerates dendrite growth, especially when distributed finely through the microstructure. The control of these microstructural features through carbide‐based synthesis has enabled substantial improvements in dendrite resistance, opening the opportunity to further fine‐tune such features for optimized performance.

## 2 Results and Discussion

**Figure 1:**
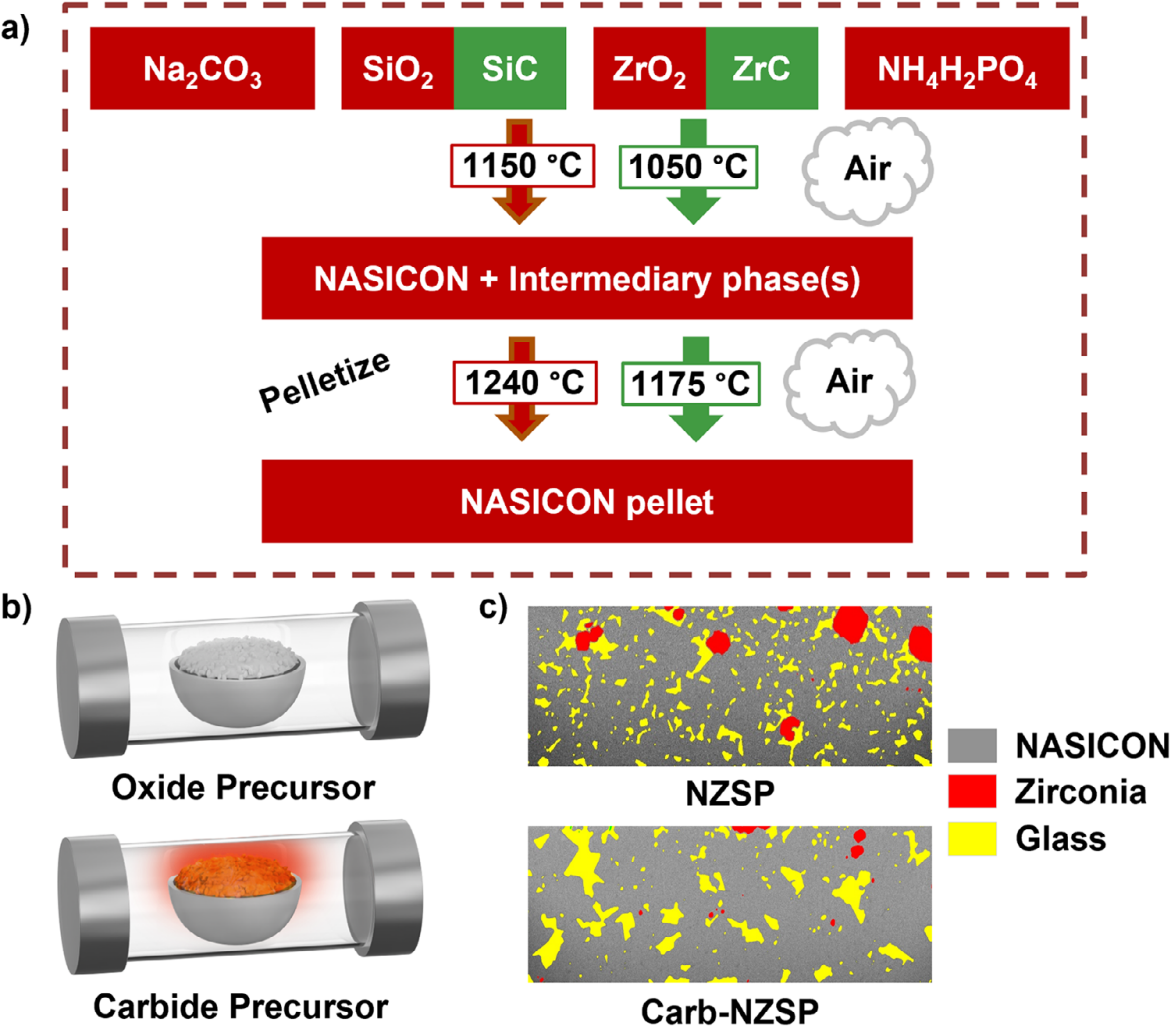
Overview of the synthesis process and resulting microstructure. a) Baseline NZSP (red) and Carb‐NZSP (green) reaction pathway. b) Schematic comparison of the baseline reaction setup and exothermic decomposition of the carbide precursor during annealing. c) Corresponding SEM/FIB cross sections with phase composition overlay.

As detailed in the Experimental Section, both the intermediate phase and the final compacts of Na_3_Zr_2_Si_2_P_1_O_12_ were synthesized using a conventional solid‐state route, except that SiO_2_ and ZrO_2_ precursors were replaced with SiC and ZrC.^[^ ^30^, ^31^, ^73^ ^]^ **Figure** **1** illustrates the schematic procedure for synthesizing carbide precursor‐based (Carb‐NZSP) solid electrolytes and the baseline oxide precursor (NZSP) specimens. Samples were mechanically mixed, annealed in air, ball milled, pressed into pellets, and sintered in air. In principle, the carbide precursor reacts in the presence of oxygen, releasing heat during the synthesis step, accelerating the synthesis process, and homogenizing the microstructure. Particle size analysis was conducted on both oxide and carbide‐based precursor powders, with results presented in Figure S1a–c (Supporting Information). The analysis revealed that both powders have a similar average particle size of ≈0.75 µm. Thermogravimetric analysis and differential scanning calorimetry (TGA‐DSC) analysis were performed on precursor mixtures for both oxide‐ and carbide‐based batches, as well as pure ZrC and SiC powders, with the results presented in Figure S2 (Supporting Information). Up to 600 °C, both precursor mixtures exhibit similar behavior, including small endothermic DSC peaks below 150 °C. A significant mass loss occurs ≈100 °C, accompanied by an endothermic peak, followed by additional mass loss between 300 °C and 600 °C. As shown in Figure S2a–d (Supporting Information), with the carbide precursor mixture, a distinct exothermic peak is observed ≈610 °C, which corresponds to the decomposition of ZrC. This exothermic event is absent in the oxide precursor mixture, which shows no sharp DSC peaks beyond 600 °C and minimal mass loss at higher temperatures. A small exothermic peak near 1100 °C in the carbide‐based precursor is absent in the oxide‐based precursor. Since this peak is not observed with pure SiC or ZrC, the nature of this peak remains unclear. The results demonstrate the exothermic decomposition of the precursor mixture, resulting from the addition of the ZrC and SiC. The proposed net chemical reactions during the procedure are presented in Equations 1 and 2 for Carb‐NZSP based and baseline NZSP, respectively. Calculated enthalpies of reaction for Carb‐NZSP and NZSP give ΔH_rxn_ = −5592.6 kJ mol^−1^ and ΔH_rxn_ = +49.3 kJ mol^−1^, respectively.^[^ ^74^ ^]^

(1) $$\begin{array}{c} 1.5{\text{Na}}_{2}{\text{CO}}_{3}+2\text{SiC}+2\text{ZrC}+{\text{NH}}_{4}H_{2}{\text{PO}}_{4}+8O_{2}\to \\ {\text{Na}}_{3}{\text{Zr}}_{2}{\text{Si}}_{2}{\text{PO}}_{\text{12}}+\text{5.5}{\text{CO}}_{2}\text{+1.5}H_{2}O+{\text{NH}}_{3} \end{array}$$

(2) $$\begin{array}{c} \text{1.5}{\text{Na}}_{2}{\text{CO}}_{3}+2{\text{SiO}}_{2}+2{\text{ZrO}}_{2}+{\text{NH}}_{4}H_{2}{\text{PO}}_{4}\to \\ {\text{Na}}_{3}{\text{Zr}}_{2}{\text{Si}}_{2}{\text{PO}}_{\text{12}}+\text{1.5}{\text{CO}}_{2}+\text{1.5}H_{2}O+{\text{NH}}_{3} \end{array}$$

**Figure 2:**
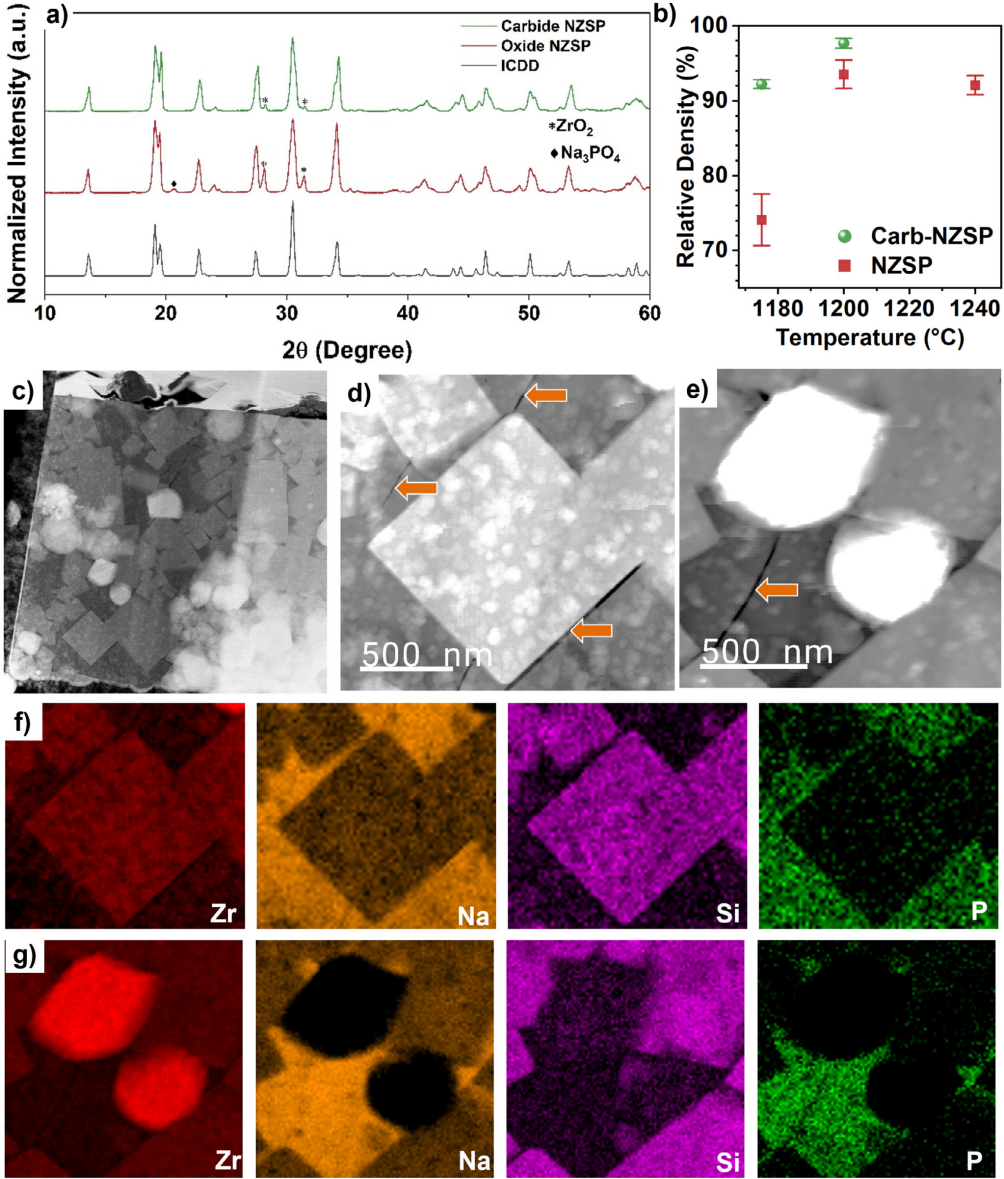
Characterization of the as‐synthesized pellet. a) XRD patterns of the sintered Carb‐NZSP and baseline NZSP pellets compared to the ICDD reference PDF#00‐035‐0412. b) Relative density of pellets sintered at various temperatures. c) Cross‐sectional cryo‐ADF‐STEM image of a Carb‐NZSP lamella. d, e) Magnified ADF‐STEM images of the two distinct regions identified in (c). f, g) cryo‐STEM‐EELS maps of the corresponding STEM images d) and e), respectively. Orange arrows show cracks in the glassy phase, as discussed in the text.

The phase purity of the NZSP compacts was evaluated using Powder X‐ray diffraction (PXRD), and the results are shown in **Figure** **2**a. Both compacts exhibit a highly crystalline NZSP phase, which aligns well with the reference pattern PDF#00‐035‐0412 (space group: C2/c), confirming successful NZSP phase formation. The sharp and well‐defined diffraction peaks indicate the high crystallinity of the synthesized NZSP compacts. Rietveld refinement of the X‐ray diffraction data is presented in Figure S3 (Supporting Information). The refined lattice parameters, atomic positions, and site occupancies for the Carb‐NZSP and baseline NZSP are presented in Tables S1–S3 (Supporting Information). Lattice parameters are near reported literature values for monoclinic NZSP.^[^ ^75^ ^]^ A notable difference between the two diffraction patterns is in the peaks associated with the secondary phases. Compacts prepared from oxide‐based precursors display prominent peaks corresponding to both tetragonal and monoclinic ZrO_2_, a common impurity in NZSP synthesized via the conventional solid‐state route. This signals incomplete incorporation of Zr during the synthesis process. The oxide based NZSP also displays an impurity peak of Na_3_PO_4_, which again has been reported as a common impurity in the synthesis of NZSP.^[^ ^76^ ^]^ Both the zirconia particles and a glassy phosphate at the grain boundaries are known secondary phases in NZSP synthesized by solid‐state routes, resulting from incomplete precursor reactions.^[^ ^77^ ^]^ In contrast, the PXRD pattern of carbide‐based compacts shows significantly weaker zirconia (ZrO_2_) peaks, indicating a reduction in impurity levels. This improvement can be attributed to the exothermic nature of the carbide precursor, which enhances the reaction kinetics for the formation of the targeted NZSP phase and the concurrent compact densification.

The exothermic reactivity of the carbide precursor enables sintering at lower temperatures, while maintaining a compact density comparable to that of baseline NZSP. Figure 2b plots the compact density versus sintering temperature for NZSP and Carb‐NZSP, highlighting the differences in densification behavior. Carb‐NZSP achieves higher compact density at 1175 °C and 1200 °C. The improved sinter density suggests that the Carb‐NZSP compacts have significantly less internal porosity than the baseline NZSP. Figure S4a,b (Supporting Information) presents digital photographs of roughly polished surfaces of Carb‐NZSP and NZSP, respectively. It can be observed that the Carb‐NZSP contains fewer macroscopic flaws on the sintered surface than the baseline. Figure S4c–h (Supporting Information) presents secondary electron images of large flaws (10–50 µm) found on polished fracture surfaces of Carb‐NZSP and NZSP pellets. The baseline NZSP pellets appear to have larger flaws in the bulk, in agreement with the lower bulk densities observed. Figure S5 (Supporting Information) shows the fractured cross‐sectional SEM images of Carb‐NZSP and baseline NZSP. The presence of unreacted zirconia (ZrO_2_) particles is particularly more pronounced in NZSP. As shown in Figure S5 (Supporting Information), the SEM images and energy dispersive X‐ray spectroscopy (EDX) maps demonstrate a higher zirconia concentration NZSP compared to Carb‐NZSP. To further examine the chemical composition of these impurity phases, cryogenic scanning transmission electron microscopy and electron energy loss spectroscopy (cryo‐STEM‐EELS) were employed.

Figure 2c–e presents cryo‐STEM images of Carb‐NZSP composite, revealing distinct structural features and compositional variations within the sample. Figure 2f,g display the EELS maps associated with the STEM images shown in 2d and 2e, respectively. The rectangular crystallite sections are relatively enriched in zirconium and silicon, identifying them as the primary NZSP phase. In contrast, the darker glassy matrix surrounding these crystallites exhibits elevated concentrations of phosphorus and sodium, identifying the secondary glassy phase not detectable by PXRD. As expected, the zirconia particles are enriched in zirconium and deficient in silicon and sodium. Figure S6 (Supporting Information) shows site‐specific analysis demonstrating the presence of a Na K edge in both the NZSP crystallite and the glass region, but not within the zirconia crystallite. The glassy region also displays cracking in multiple areas (as indicated by the arrows), indicative of its brittle mechanical properties, even relative to NZSP. Bright, crystalline particles of zirconia are also observed, appearing as distinct, high‐contrast features within the microstructure.

**Figure 3:**
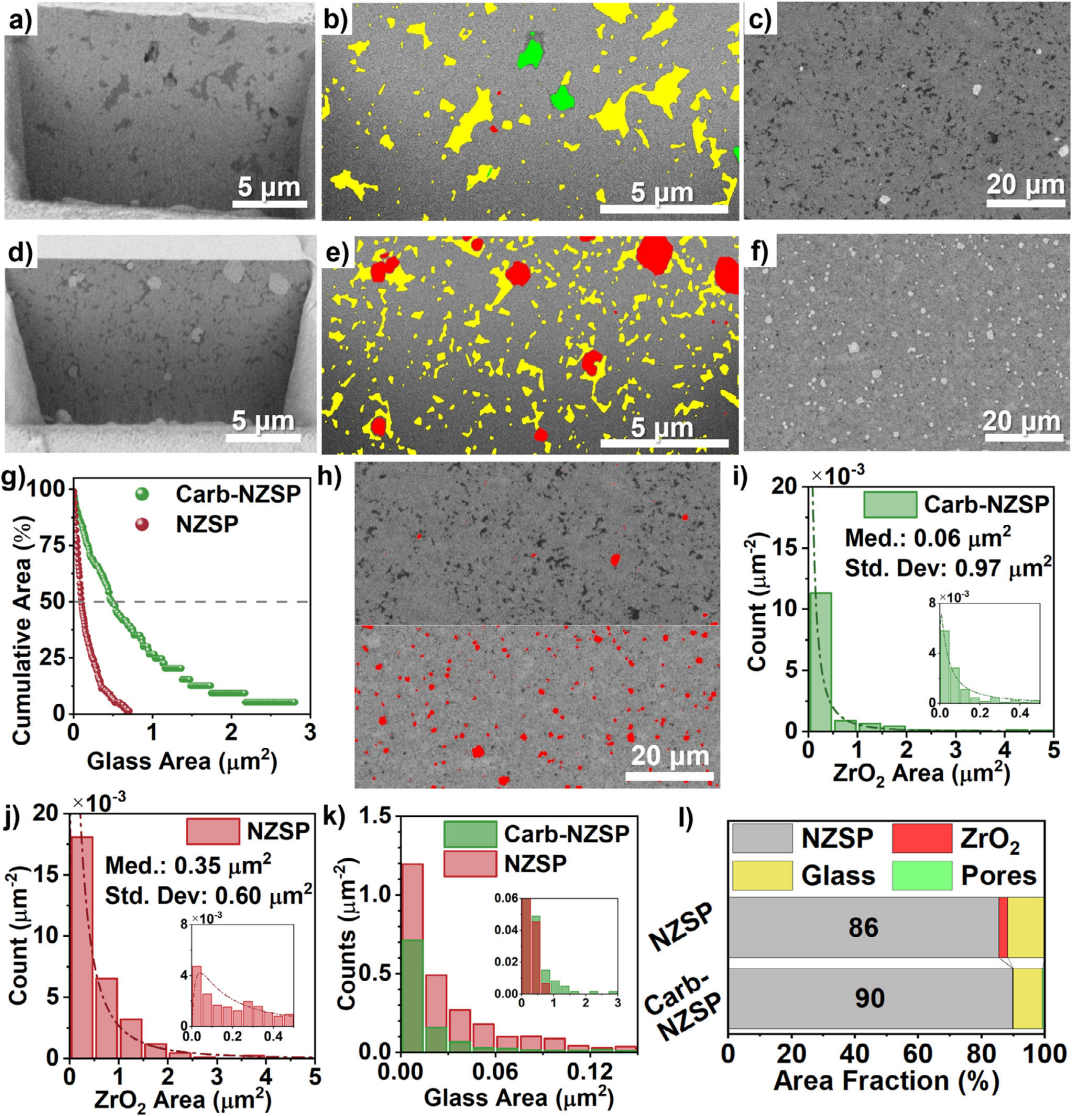
a,d) Representative FIB SEM cross sections of Carb‐NZSP and baseline NZSP, respectively. b,e) Corresponding stereology analysis maps of zirconia particles (red), glassy phase (yellow), and porosity (green). c,f) SEM images of mechanically polished fracture surfaces for Carb‐NZSP and NZSP, respectively. g) Comparison of the cumulative area distribution of the glassy phase in both materials. h) Example of stereology analysis for the zirconia in (top) Carb‐NZSP and (bottom) NZSP specimens. i,j) Distribution of zirconia particle cross sectional areas for Carb‐NZSP and NZSP, respectively. k) Distribution of glass phase cross sectional areas for both materials. l) Summary of the stereology‐derived area fraction of the four phases in Carb‐NZSP and NZSP.

To quantify the distribution and composition of the Carb‐NZSP and baseline NZSP, SEM‐FIB was utilized in combination with quantitative stereology analysis based on NIH ImageJ. **Figure** **3**a–f provide high magnification SEM‐FIB cross‐sectional images, and low magnification fractured polished cross‐sectional images of the compacts. Phase quantification maps are presented in Figure 3b–e for Carb‐NZSP and NZSP, respectively. Overall, the Carb‐NZSP compact cross‐sections exhibit 90% ± 2% crystalline NZSP phase with the remainder being impurity phases, whereas oxide‐based NZSP cross‐sections contain 86% ± 2% crystalline NZSP phase. Carb‐NZSP exhibits a zirconia volume fraction of 0.2% ± 0.3%, whereas baseline NZSP shows a much higher value of 3% ± 1%. Quantification of the glass phase reveals a volume fraction of 9.1% ± 2.1% for Carb‐NZSP, compared to 11.7% ± 1.2% in the baseline. These results are illustrated in Figure 3l. Due to the limited sample area available by SEM‐FIB, variations in the compact porosity are not statistically significant. No pores are observed in the NZSP images, with Carb‐NZSP pores making up 0.9% ± 0.6%. A summary of the cross‐sectional phase composition is presented in Table S4 (Supporting Information), with all FIB sections presented in Figure S7 and S8 (Supporting Information).

To probe the variability of cross‐section composition throughout the bulk of the pellets, additional FIB sections were cut from various regions in both Carb‐NZSP and NZSP pellets, as shown in Figure S9 (Supporting Information). FIB section regions were selected from near the pellet center, face, and edge, as shown in Figure S9a (Supporting Information). Results shown in Figure S9b–m (Supporting Information) indicate minor variations in cross section composition and phase distributions between various regions of each pellet. The general findings are further supported by the fractured, polished cross section analysis, as shown in Figure 3e and Figures S10a–f (Supporting Information). On the fracture surfaces, the measured zirconia volume fraction is 0.6% ± 0.2% for Carb‐NZSP, compared to 3.4% ± 0.4% for NZSP.

To further elaborate on variations in zirconia volume fractions, stereology analysis of particle size distributions for zirconia in the two NZSP types was conducted using the same two approaches: FIB milled and fractured, polished cross‐sections. Analysis of multiple sample areas was performed to reduce local microstructural effects. Representative results are presented in Figure 3I,j and Table S5 (Supporting Information), with additional images shown in Figure S10 (Supporting Information). According to Figure 3i, for Carb‐NZSP fractured cross‐sections, the median zirconia particle area was found to be 0.06 µm^2^, with an average area of 0.42 ± 0.13 µm^2^. In comparison, NZSP fractured cross‐sections demonstrated a significantly larger median area of 0.35 µm^2^, and an incrementally larger average zirconia particle area of 0.53 ± 0.01 µm^2^, per Figure 3j. The dramatic reduction in total zirconia volume fraction (measured as area fraction in 2D) and number density of zirconia particles in Carb‐NZSP indicates more complete incorporation of zirconium element into the NZSP matrix.

A similar stereological analysis was conducted for the glassy phase in both Carb‐NZSP and baseline NZSP compacts. While the difference in the overall volume fraction of the glassy phase between the two materials is not particularly significant, the distribution of this phase within the compact varies substantially. As shown in Figure 3b and Figure S7 (Supporting Information), the glassy phase in Carb‐NZSP is more agglomerated. In contrast, the glass phase in baseline NZSP is relatively dispersed, per Figure 3e and Figure S8 (Supporting Information). The distribution of glass section areas is presented in Figure 3k, with the cumulative area fraction distribution of the glass phase presented in Figure 3g. In the Carb‐NZSP samples, 50% of the total glass phase accumulates in individual sections larger than 0.48 µm^2^, compared to 0.10 µm^2^ for the baseline. While none of the baseline images reveal a glass section larger than 1.0 µm^2^, more than 26% of the glass in the carbide sample can be found in areas larger than 1.0 µm^2^. A summary of the size distribution of glass sections is presented in Table S6 (Supporting Information). The section perimeter to area ratio, (L_A_)_g_ is related to the surface area to volume ratio of the total glass phase. For the smaller, more dispersed baseline NZSP, the glass perimeter to area ratio, (L_A_)_g_, is 18.77 ± 0.64 µm^−1^, while for Carb‐NZSP glass section (L_A_)_g_ is 11.61 ± 0.72 µm^−1^. This difference indicates the concentration of glass content into large volumes for Carb‐NZSP, resulting in it being more isolated.

The grain boundary glassy phase has been described in literature as low melting point mixture of the type Na_2_O‐P_2_O_5_‐SiO_2_ solid.^[^ ^77^, ^78^ ^]^ Kimura et al. have shown that excess glass phase reduces ionic conductivity between NZSP grains, and its removal via hot pressing increases grain boundary conductivity. Quantitative characterization of the extruded glass composition, by WDS showed a glass rich in Si and P.^[^ ^65^ ^]^ However, variations in the Na_1+x_Zr_2_Si_x_P_3‐x_O_12_ composition, as well as the use of excess Na and P during synthesis further complicate comparisons between research groups. A high temperature heat treatment of the NZSP surface has been shown to produce a Na and P rich interphase. It is known to reduce interfacial resistance and increase molten Na wettability.^[^ ^76^, ^77^, ^79^ ^]^ The similar elemental composition between the grain boundary glass and surface glass phases suggests that the two secondary phases may be similarly sodiophilic and would therefore “attract” growing sodium metal during electrochemical cycling. As long as there is an electrical path back to the current collector surface, the dendrite will advance along the path where pores and the glassy phase are concentrated.

This altered microstructure is expected to have significant impact on the mechanical properties of the sintered ceramic compacts. Figure S11 (Supporting Information) presents Vickers hardness micro indentation testing results for both NZSP and Carb‐NZSP. The average hardness of NZSP and Carb‐NZSP are 4.7 and 6.1 GPa, respectively. The higher micro‐hardness of Carb‐NSZP is attributed to the higher relative density and less glassy phase compared with the baseline. In sintered ceramic compacts, pores left over due to incomplete densification are present between the primary sintered grains/particles, even when grain growth occurred during densification.^[^ ^80^ ^]^ In sintered NZSP compacts, dendrite tolerance is promoted by reducing the volume fraction of pores, i.e., by increasing compact density. For a range of electrolytes, pores are known to promote dendrite intrusion by acting as reservoirs where metal is electrodeposited, leading to localized stress build up and fracture of the electrolyte, and subsequent electrodeposition onto the exposed surfaces.^[^ ^81^, ^82^, ^83^, ^84^, ^85^ ^]^

For garnet‐based solid electrolytes both the size and shape distribution of pores resulting from varied sintering conditions showed strong correlation with the resultant ionic conductivity.^[^ ^86^ ^]^ In Carb‐NZSP, the distribution and content of the glass phase along grain boundaries will affect the ionic conductivity mechanisms through either the bulk crystallites or through grain boundaries. However further analysis is needed to fully establish such differences in conduction mechanism. It is logical that less porosity within the Carb‐NZSP microstructure (higher compact density) will make it more dendrite resistant and more ionically conductive, providing one reason why Carb‐NZSP is electrochemically superior to the baseline.^[^ ^86^ ^]^ An analogous argument may be made regarding the sodiophilic glassy phosphate phase that coats the NZSP grains. Reducing the connectivity (i.e., the distribution, the percolation path) of the glassy phosphate around the primary NZSP grains will aid electrochemical stability. Percolated throughout the bulk of the electrolyte, sodiophilic glass may actually serve as a wetting template that steers the dendrite, especially if the glass easily cracks and/or is in contact with pores. Carb‐NZSP contains isolated (vs nearly continuous) pockets of the glassy phase and is substantially more dense, both microstructural features promoting electrochemical stability.

**Figure 4:**
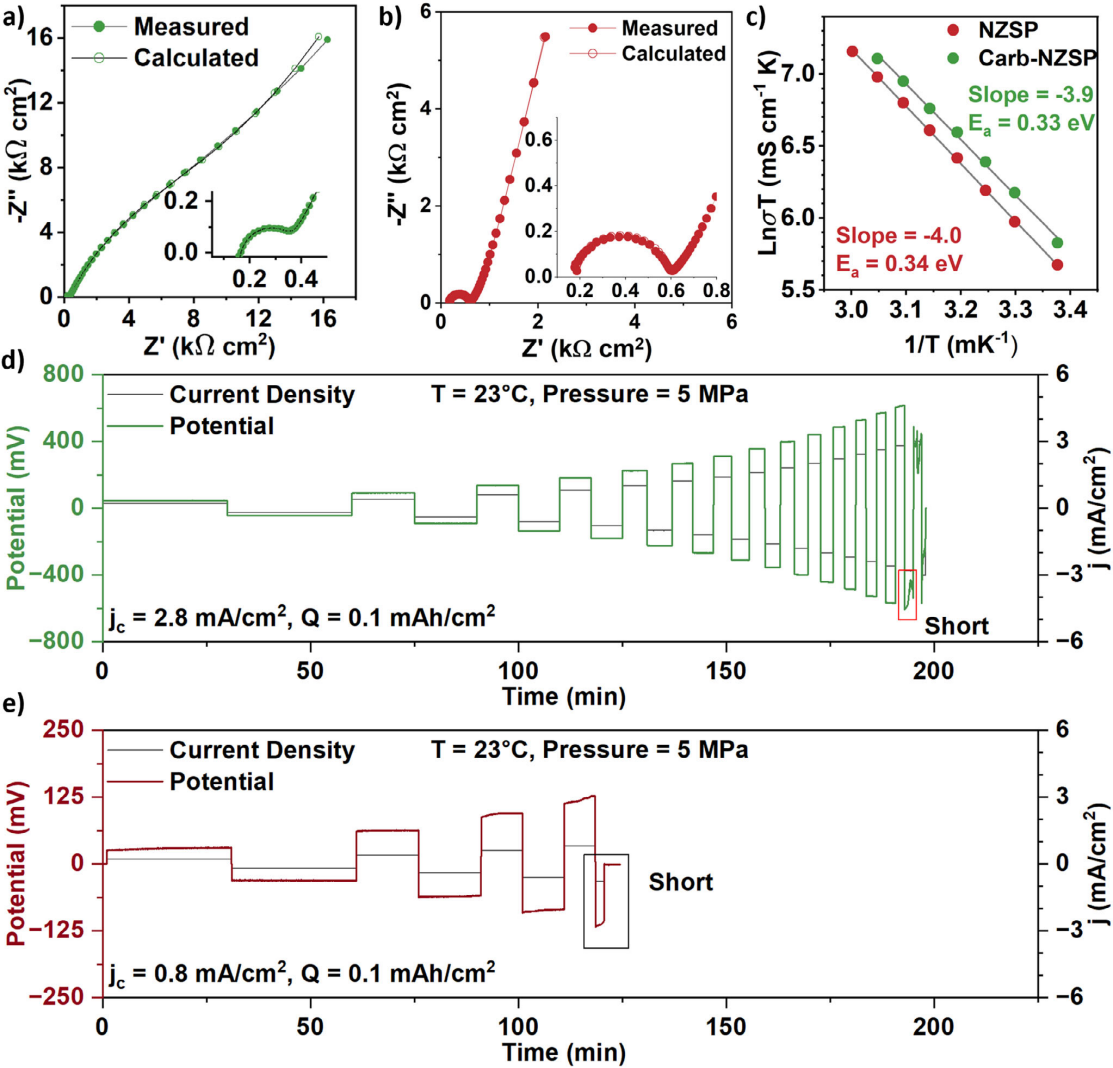
a,b) Measured and modelled EIS Nyquist plots (using W blocking electrodes) of Carb‐NZSP and NZSP compacts, including higher magnification insets of the high frequency regime. c) Arrhenius plot comparing the activation energy for sodium transport in both materials. d,e) Representative variable current critical current density (CCD) tests, with deposition/dissolution time varied to achieve constant capacity of 0.1 mAh/cm^2^ per cycle, performed at room temperature.

Detailed electroanalysis was performed to understand the influence of microstructure on the electrochemical performance of the electrolytes. **Figure** **4**a,b presents the Nyquist plots obtained from room‐temperature (23 °C) potentiostatic electrochemical impedance spectroscopy (PEIS) experiments conducted on Carb‐NZSP and NZSP, respectively. In these measurements, tungsten (W) served as the Na‐blocking electrode on each side of the compact. The PEIS experiments were performed over a frequency range of 5 MHz to 1 Hz, with a logarithmic step size distribution. To ensure accuracy, PEIS experiments were conducted on multiple sets of compacts.

Both compacts exhibited a series resistance and a prominent semicircle within the frequency range of 5 MHz to 100 kHz, corresponding to the bulk and grain boundary resistances, respectively. The Carb‐NZSP and baseline NZSP displayed on par average ionic conductivity of 1.08 ± 0.09 mS and 1.02 ± 0.06 mS cm^−1^, respectively. Those results are shown in Figure S12a (Supporting Information). Further characterization involved measuring sodium ionic conductivity at seven different temperatures: 23, 30, 35, 40, 45, 50, 55, and 60 °C. The resulting data were plotted as the logarithm of the product of ionic conductivity and temperature versus the inverse of temperature for both types of NZSP compacts. Those results are provided in Figure 4c. The activation energy (E_a_) for sodium ion transport was determined using the Arrhenius equation.^[^ ^20^ ^]^

(3) $$\sigma T=\sigma_{0}e^{\left(-E_{a}/k_{B}T\right)}$$

where σ is the ionic conductivity, σ_0_ is the pre‐exponential factor, T is the temperature, k_B_ is the Boltzmann constant, and E_a_ is the activation energy. Over the tested temperature range, the total activation energy for Carb‐NZSP and NZSP was also similar, being 0.33 and 0.34 eV. As will be demonstrated next, the major difference between Carb‐NZSP and baseline NZSP is in the Na electrodeposition/dissolution characteristics, specifically in their resistance to dendrites.

Cryogenic SEM‐FIB milling and galvanostatic electrochemical experiments were conducted to investigate the sodium interfacial electrodeposition/dissolution behavior of the Carb‐NZSP and NZSP compacts. Details of the symmetric cell and half‐cell fabrication are provided in the experimental section. Potentiostatic electrochemical impedance spectroscopy (PEIS) was performed on multiple symmetric cells to determine the average interfacial impedance values. Figure S12b,c (Supporting Information) displays the average calculated interfacial impedance values and Nyquist plots of representative symmetric cells fabricated with the two electrolytes. Carb‐NZSP demonstrated an average interfacial impedance of 19.3 ± 4.6 versus 24.8 ± 8.6 Ω⋅cm^2^ for NZSP. There is incrementally lower average impedance and less sample‐to‐sample variation with Carb‐NZSP electrolyte. This is attributed to Carb‐NZSP enabling a more chemically and geometrically uniform Na‐SSE interface, in‐turn due to a lower phosphate, zirconia, and porosity content of the mating surface.

To evaluate long‐term cycling performance, symmetric cells were cycled at constant current densities of 0.1, 0.5, and 1.0 mA cm^−2^ with 1‐h intervals per half cycle, corresponding to a sodium electrodeposition/dissolution capacity of 0.1, 0.5, and 1.0 mAh cm^−^^2^, respectively. Figure S13a (Supporting Information) presents the 0.1 mA cm^−2^ results for a Carb‐NZSP symmetric cell, which exhibited an average overpotential of 20 mV and stable cycling behavior for over 1000 cycles (≈2000 h) without any signs of short‐circuiting. The corresponding NZSP symmetric cell is presented for comparison in Figure S13b (Supporting Information). This baseline cell also demonstrated stable cycling for over 1000 cycles (≈2000 h), with an average overpotential of 30 mV, which increases gradually over time. Figure S13c,d (Supporting Information) presents the 0.5 mA cm^−2^ cycling results for 180 h of stable cycling with nearly constant overpotentials ≈100 mV. Substantial differences arise from the 1.0 mA cm^−2^ cycling results, in which the NZSP cell short circuits in only 8.5 h, while the Carb‐NZSP demonstrates stable cycling over 80 h, with polarization runaway developing between 100 and 180 h. It may be concluded, therefore, that there are no major differences in the electrochemical behavior of Carb‐NZSP versus baseline NZSP at a low current density/capacity. However, as current densities increase, the dendrite tolerance of Carb‐NZSP becomes increasingly important to electrochemical performance.

To gain further insight into dendrite growth behavior in the two types of NZSP materials, critical current density (CCD) experiments were conducted on symmetric cells employing Carb‐NZSP and NZSP. In these experiments, galvanostatic cycling was performed using a constant electrodeposition/dissolution capacity of 0.1 mAh cm^−2^ per step. The current density was incrementally increased by 0.2 mA cm^−2^ per cycle, starting from 0.2 mA cm^−2^, until the onset of electrical shorting. As shown in Figure 4d and Figure S14a,b (Supporting Information), the Carb‐NZSP symmetric cells achieved a CCD of 3.1 ± 0.8 mA cm^−2^. In contrast, the baseline NZSP symmetric cells short‐circuited at a much lower CCD of 1.0 ± 0.7 mA cm^−^^2^. This result is shown on Figure 4e and Figure S14c,d (Supporting Information). The significantly higher CCD and extended cycling stability of the Carb‐NZSP versus baseline NZSP highlight its enhanced resistance to dendrite‐induced failure. Table S10 (Supporting Information) compares the CCD and ionic conductivity values Carb‐NZSP to the state‐of‐the‐art literature for NASICON. It may be observed that overall Carb‐NZSP is quite promising, especially considering that the current synthesis method follows a conventional solid‐state synthesis method, rather than a sol‐gel or pressure‐assisted route.

In addition to dendritic growth behavior, Na‐NZSP interfaces are susceptible to contact loss and associated current constriction. Cryogenic SEM‐FIB was employed to explore the Na‐NZSP interface in the pristine and post‐mortem states. Figure S15 (Supporting Information) shows two Na‐NZSP symmetric cell interfaces, one prior to being cycled, and one after cycling per the above CCD protocol until it short circuited. The cycled interface is of a cell that short circuited. The image is of a region without a dendrite visible. The pristine interface displays relatively continuous contact between Na metal and NZSP. However, the cycled interface is characterized by additional porosity in the Na metal and accompanying delamination. These results suggest that at this low current density/capacity, interfacial contact loss is responsible for the increase in overpotential over time. Cycling induced interface contact loss would occur prior to any electrical short‐circuiting events and is expected to accelerate the process through current constriction effects. This agrees with the literature interpretation that interfacial pores lead to heterogeneous electrodeposition/dissolution at the remaining contact points, with the true current density surpassing the geometric current density.^[^ ^87^, ^88^, ^89^, ^90^, ^91^, ^92^ ^]^ The phenomenon of contact loss is documented in literature. Ortmann et al. utilized in situ XPS, STEM‐EDX, cryo‐SEM‐FIB, and PEIS to explore the effects of time, temperature, and pressure on the Na|NASICON interface.^[^ ^93^ ^]^ The results demonstrate that reactive SEI formation is minimal, while contact loss is substantial. This interfacial contact loss and current constriction is expected to be active in both Carb‐NZSP and NZSP, with factors such as post‐fabrication interfacial roughness and incomplete wetting of Na metal being important determinants of cycling life.

To further understand the role of microstructure in electrochemical stability, aggressive current density – capacity cycling was performed until failure via short circuit on several cell configurations. Symmetric cells were cycled with 5 activation cycles at 0.1 mA cm^−2^ and 0.1 mAh cm^−2^, followed by 0.5 mA cm^−2^ at 0.5 mAh cm^−2^ until short circuiting. In addition, cycling of asymmetric half‐cells of both NZSP and Carb‐NZSP was performed, with those specimens analysed by cryo‐FIB as well. The symmetric cells were cycled at room temperature, while the half‐cells were cycled at 55 °C. The half‐cells were electrodeposited with a current density of 0.1 mA cm^−^^2^ for 1 h for the NZSP cell, while the Carb‐NZSP was cycled at 0.5 mA cm^−2^ for 5 cycles followed by 1.0 mA cm^−2^ until failure. Each electrodissolution was performed at the same current density as the previous electrodeposition to a 1 V limit. The electrochemical cycling data for the NZSP symmetric cell and the NZSP and Carb‐NZSP half‐cells are provided in Figure S16a–d, (Supporting Information), respectively.

**Figure 5:**
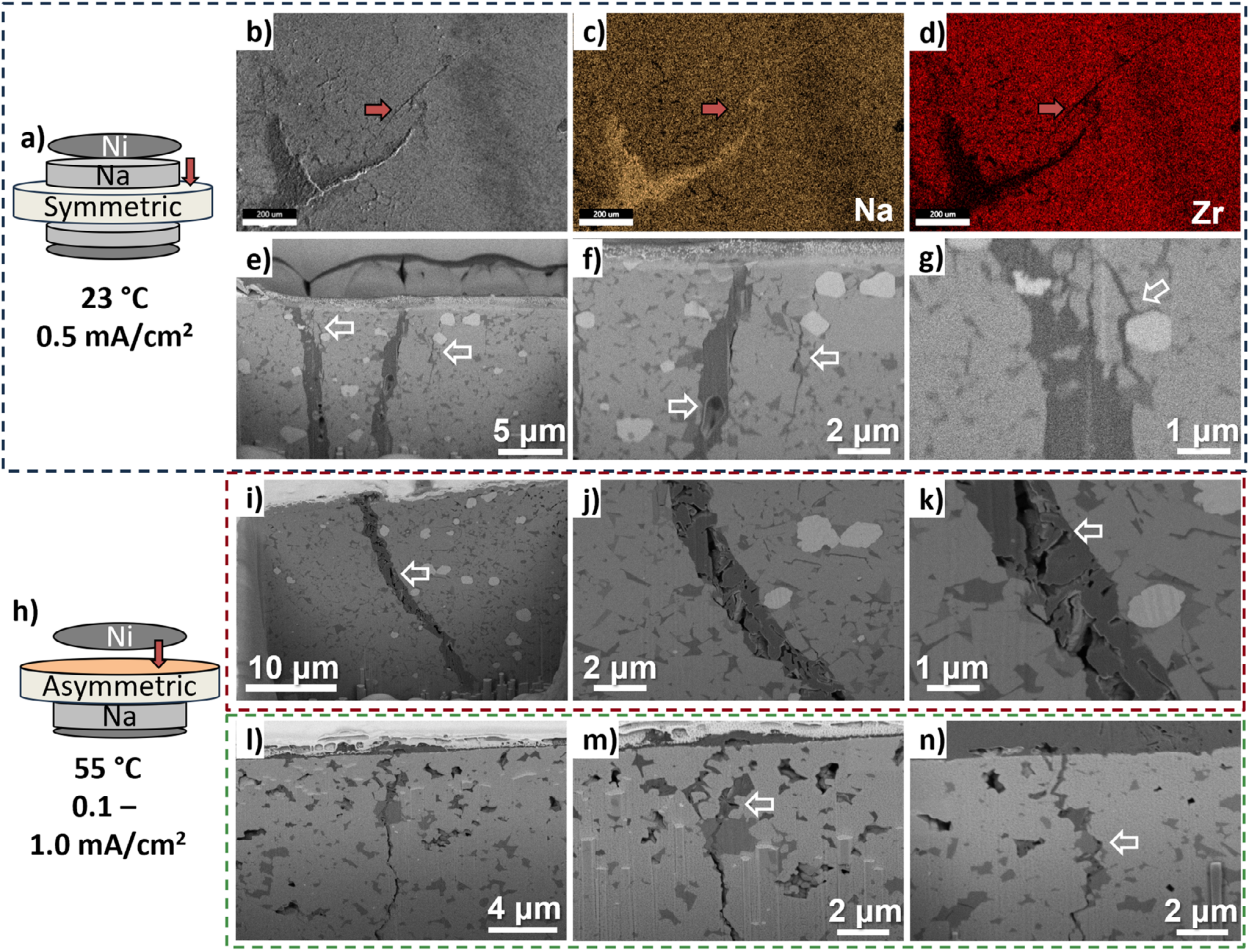
Cryo‐FIB analysis of dendrites in NZSP and Carb‐NZSP. a) Schematic of the symmetric cell employed for analysis, shown in b–g), tested at room temperature. (b–g) Secondary electron top‐down SEM image corresponding Na and Zr EDX maps of a sodium metal dendrite observed in a crack on the surface of the NZSP cell. (e–g) Backscattered electron SEM images taken from FIB cross sections of the same dendrite, presented at increasing magnification. h) Schematic of the asymmetric half‐cell configuration employed for analyses of i–k) NZSP and l–m) Carb‐NZSP, both tested at 55 °C.

**Figure** **5** illustrates top‐down and FIB cross sectional analysis of Na metal dendrite in NZSP cells that were cycled until short circuit failure. A schematic of the experimental setup for symmetric cells is presented in Figure 5a, with the red arrow point to the region that was analyzed first in top‐down and then in FIB cross‐section. Figure 5b–d shows the top‐down view secondary electron image of the Na metal dendrite and corresponding Na (orange) and Zr (red) EDX maps. Additional EDX maps are presented in Figure S17a–e (Supporting Information). It may be observed that the sheet‐like Na metal dendrite is present in the proximity of the crack within the NZSP (arrowed). Per the secondary electron image and the Na and Zr maps, the dendrite's thickest portion is located outside the crack. At its leading edge (sharpest point), the dendrite penetrates into the crack, filling it and thereby generating an enhanced Na signal. After top‐down analysis, the arrowed region of NZSP was FIB milled to expose the crack's interior and the associated dendrite in cross‐section. Backscattered electron SEM images were taken to differentiate various components by Z‐number (Na metal is less dense and more electrically conducting, hence appearing darker). Those results are presented in Figure 5e–g. It may be observed that the dendrite is a highly branched 2D sheet, rather than one or a series of isolated 1D filaments. The dendrite sheet contains porosity, implying that it was electrochemically active enough to allow for not only electrodeposition during cycling but also for localized electrodissolution. Numerous thinner secondary branches of the dendrite appear to emanate from the two (in the field of view) primary branches that are much thicker. Some of these secondary branches fill sharp microcracks in the NZSP. It is difficult to know whether these microcracks were present prior to cycling, or whether they were generated in situ ahead of the advancing metal due to the localized stress. However, most of the thinner dendrite branches appear to flow around and isolate fragments of NZSP as well as individual zirconia particles.

**Figure 6:**
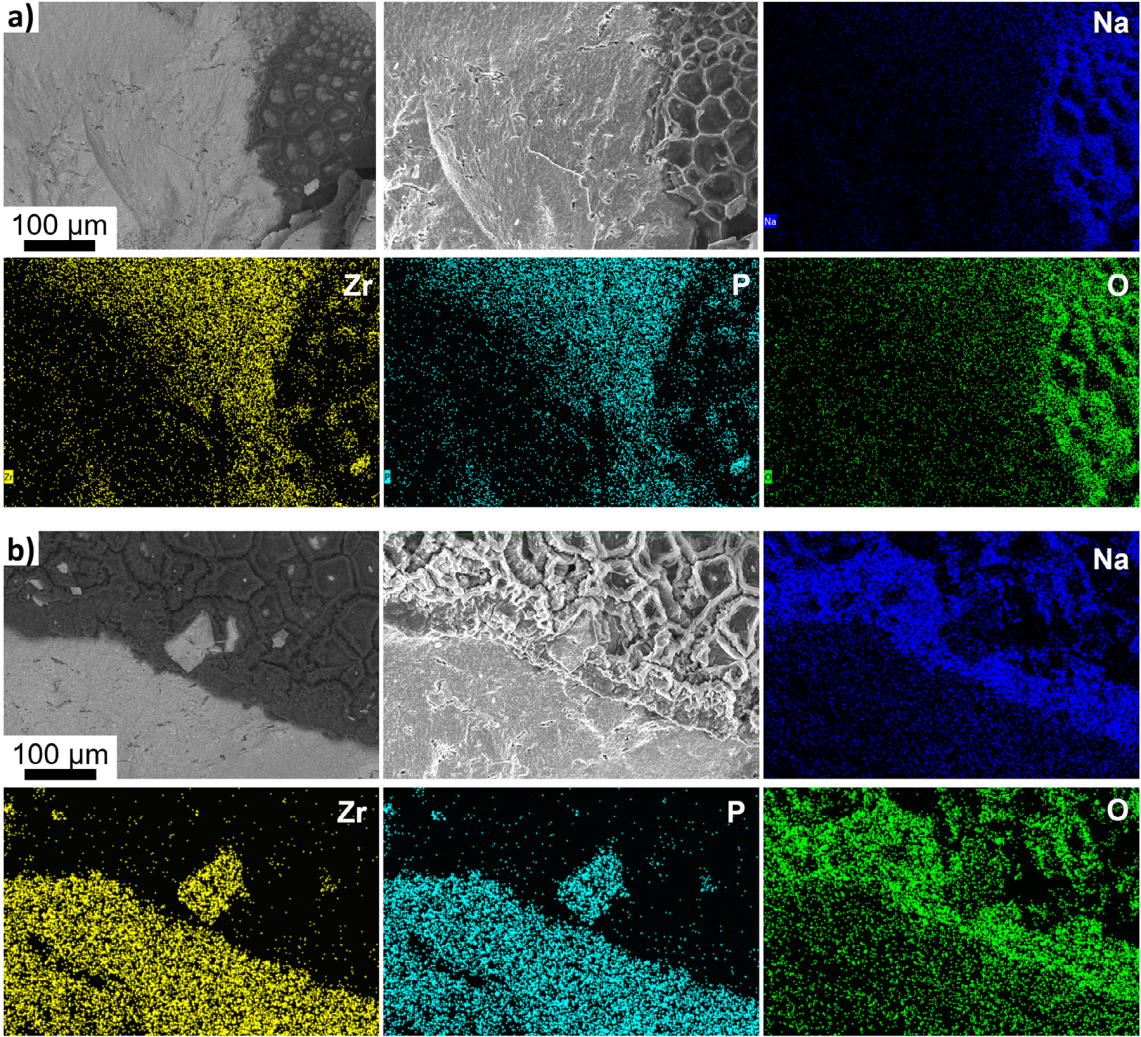
Post‐mortem backscatter and secondary SEM cross‐sectional images and corresponding EDX images of a symmetric cell employing a) Carb‐NZSP based and b) baseline NZSP based symmetric cell after a short under CCD testing (0.2 mA cm^−2^, 0.2 mA cm^−2^, 0.1 mAh cm^2^). Na (blue), Zr (yellow), P (cyan), and O (green).

Figure 5h–k and Figure S17f–m (Supporting Information) presents analysis of an NZSP short‐circuited asymmetric half‐cell, tested at 55 °C. An analogous phenomenology is revealed. Porous branched sheet‐like dendrites that engulf both the NZSP grains and the zirconia particles. Interestingly, the degree of porosity in the metal is greater in these specimens. This may be due to the elevated temperature of testing and/or due the limited reservoir of Na available for electrodissolution in a half‐cell. Figure 5l–n presents the FIB cross sections of a Carb‐NSZP asymmetric cell cycled until short circuiting at more aggressive current densities. The associated electrochemical data are shown in Figure S16d (Supporting Information). A crack through the solid electrolyte is filled with sodium metal near the surface, but appears empty ≈8 microns below the sample surface. Near the surface, the thin dendrite can be seen to deflect at large angles, likely around NASICON grains. Cryo‐FIB analysis, shown in Figure 5l–n, reveals a large section of the phosphate glass (white arrows) through which the metal dendrite penetrates, further supporting the role of the glass phase in dendritic failure. **Figure** **6**a,b provides more “macroscopic” postmortem analysis of post‐cycled Carb‐NZSP and NZSP electrolyte surfaces. For both electrolytes, dendrites accumulate within the bulk of the compacts. They display a branched 2D sheet‐like morphology, flowing around and engulfing numerous NZSP grains.

To further elucidate the role of secondary phases in dendrite propagation, a phase‐field model was applied to the NZSP‐ZrO_2_‐phosphate glass system over a variety of secondary particle sizes and quantities. The phase‐field modelling framework is comprised of a series of coupled partial differential equations that, broadly speaking, enforce conservation of electrical, chemical, and mechanical energy. The system consists of two auxiliary phase‐field variables that represent the Na metal filaments and cracks as well as several field variables that represent physical quantities such as the concentration of Na^+^ ions. The applied electric field drives Na^+^ ions down the potential gradient, leading to electrodeposition on the surface of the Na metal. As this occurs, pressure develops in the burgeoning filament, which must be balanced by stresses in the surrounding electrolyte matrix. These stresses lead to the growth of brittle cracks, which present an attractive pathway for the Na filament to continue growing.

**Figure 7:**
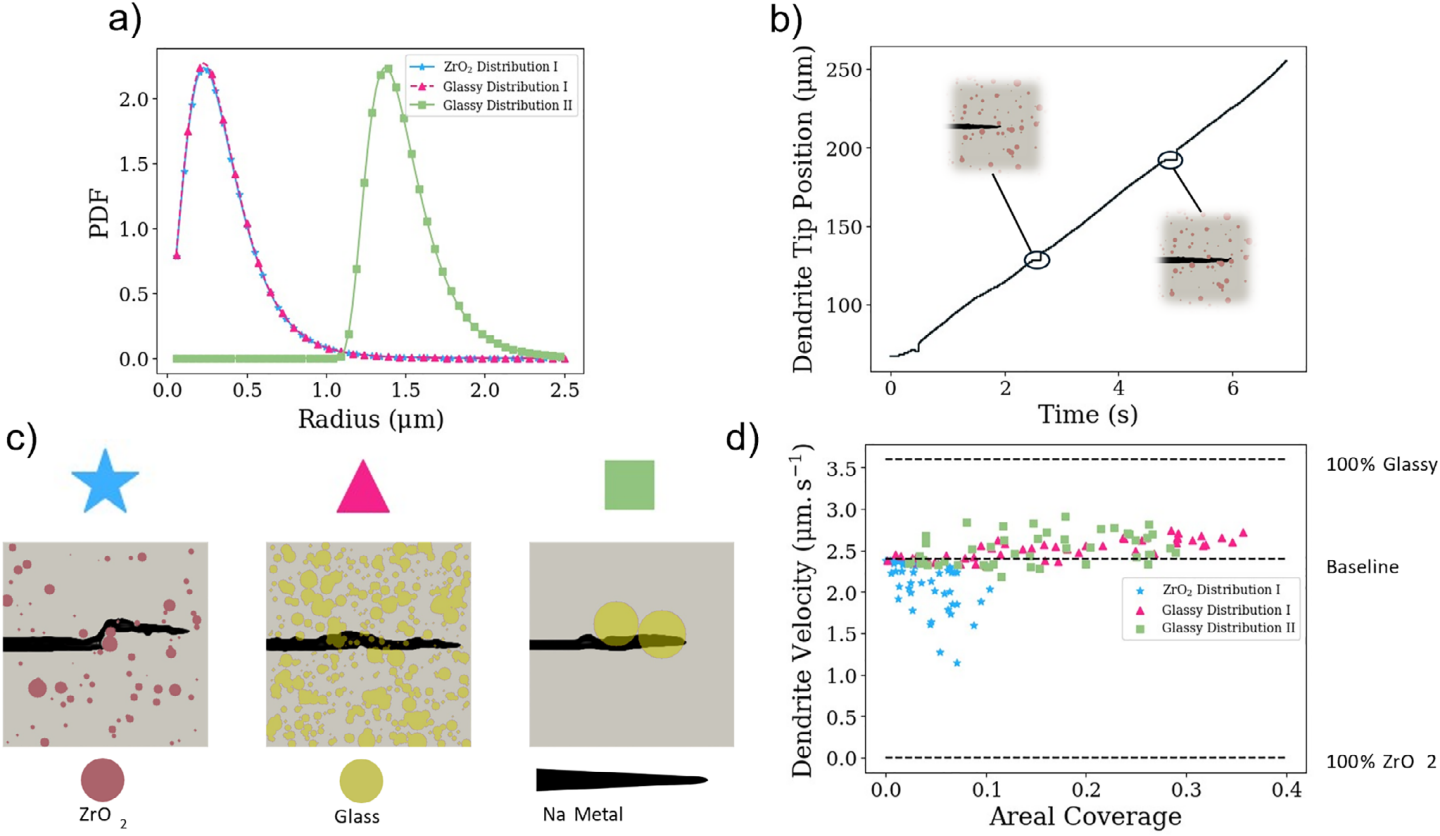
Phase‐field model parameters and results of sodium metal dendrite propagation through NZSP‐ZrO_2_ and NZSP‐phosphate glass composites. a) input distributions of particle sizes of ZrO_2_ (blue) and phosphate glass (red). b) Example resultant dendrite tip growth over time through NZSP‐ZrO_2_. c) Point‐in time model results showing dendrite interaction with zirconia and phosphate glasses of various size distributions. d) dendrite average velocity for each particle size distribution over a range of areal coverages.

**Figure** **7**a shows three sample distinct probability distributions of ZrO_2_ and glassy phosphate particles. All three distributions are derived from the log‐normal distribution. Figure 7c shows the growth of Na dendrites through microstructures comprised of secondary particle distributions from Figure 7a within an NZSP matrix. Generally, the dendrites deflect around the tougher, more Na^+^‐poor ZrO_2_ particles as the path of least resistance leads through the comparatively brittle NZSP domains. By contrast, the dendrites preferentially hew to both the bulk of the glass particles as well as NZSP‐glassy interfaces, where the material is both more brittle and more Na^+^‐rich. The dendrite passes through more particles in glassy distribution I than in glassy distribution II, solely due to the greater abundance of the particles in this distribution. One can reasonably infer that dendrites, in the most general case, will spend more time in the brittle, high Na^+^ concentration domains of distribution II than in distribution I and consequently will exhibit a larger time‐averaged velocity.

Figure 7b shows an example evolution of a Na‐dendrite tip position (measured along the *x* axis of the simulation domain) in a microstructure consisting of ZrO_2_ particles. The plateaus correspond to times when the dendrite is forced to traverse a more tortuous path around the zirconia particles, and the insets show this process. This process may happen in either the ZrO_2_ or glassy microstructures; indeed, the dendrite may deviate from the straightest path to either skirt around the tougher ZrO_2_ particles or to selectively pass through/along the surface of glassy particles. The analysis sampled 50 configurations of secondary particles (i.e., ZrO_2_ or glassy), matching distribution I (ZrO_2_ and glassy) and distribution II (solely glassy), with areal coverages ranging from 0 to 40%.

Figure 7d demonstrates the sampled velocities of dendrites in each of these microstructures. Increased abundance of ZrO_2_ particles clearly leads to a decreased dendrite velocity, while increased abundance of glassy particles leads to increased dendrite velocity. Additionally, both the ZrO_2_ distribution I and glassy distribution II velocities exhibit a larger standard deviation than the glassy distribution I velocities. The results are that for ZrO_2_ distribution I, the dendrite velocity is 2.23 µm s^−1^ with a standard deviation of 0.29 µm s^−1^. For glassy distribution I, the dendrite velocity is 2.52 µm s^−1^ with a standard deviation of 0.12 µm s^−1^. For glassy distribution II, the dendrite velocity is 2.47 µm s^−1^ with a standard deviation of 0.19 µm s^−1^.

While dendrite velocity constitutes a simple measurement, the far more difficult‐to‐capture critical current density is more germane to the operability of battery cells using these materials. Herein, we adopt a heuristic argument that dendrite velocity is inversely correlated to dendrite resistance and, consequently, the critical current density. This idea has its roots in the conclusion that dendrites will nucleate and grow faster where there is a larger thermodynamic driving force. That driving force consists of two components: (i) the ease of deposition and (ii) the dendrite's ability to create an environment conducive to deposition. The conditions that inform (i) are, among other things, the abundance of reactants (Na^+^ ions and electrons) and the magnitude of the local stresses. The latter decreases the ease of deposition, while the former increases the ease of deposition. Item (ii) specifically describes the dendrite's ability to clear space for itself by cracking the surrounding solid electrolyte matrix. This is dictated by how brittle the surrounding material is. That the dendrite velocity decreases in the ZrO_2_ subdomains and increases in the glassy subdomains indicates that the thermodynamic driving force for deposition is smaller in the former and larger in the latter. Thus, it is reasonable to expect that initiation and propagation of dendrites will occur at lower potentials and currents in microstructures that are rich with glassy particles and at higher potentials and currents in microstructures that are rich with ZrO_2_ particles. The spread in the distributions also comprises an important parameter. This most clearly manifests in glassy distribution II and ZrO_2_ distribution I. These two cases exhibit both velocities close to the baseline value at high areal coverages as well as far from the baseline at low areal coverages. From a purely stochastic perspective, the probability of a glassy particle being close to the surface of the Na anode is lower in distribution II than in distribution I. This will raise the CCD, as there will be a lower probability of favorable nucleation/cracking sites near the electrochemical reaction.

Finally, we should briefly discuss the techno‐economics of an approach based on the substitution of carbide precursors for oxide precursors. The growing demand for global energy storage and increasing interest in solid state electrolytes will necessitate advancements in manufacturing processes to achieve thin, mechanically robust, highly conductive SSEs.^[^ ^94^ ^]^ Consider the substitution demonstrated in this study, SiC for SiO_2_ and ZrC for ZrO_2_ in NASICON. Silicon carbide can be produced in a bulk high‐temperature reaction in air by the Acheson process, while ZrC requires synthesis in more inert atmospheres, such as through carbothermal reduction.^[^ ^95^, ^96^, ^97^ ^]^ The cost of SiC is relatively inexpensive at around USD$800/ metric ton, and would not drastically affect the cumulative cost of production. The price of ZrC powder at an industrial scale (tons) is not readily available, although both ZrO_2_/ZrC should be more expensive than SiO_2_/SiC. The reduced sintering temperature enabled by the use of carbide precursors will lower processing costs by reducing the energy demand and prolonging the lifetime of the heat treatment equipment. In parallel, the increased compact density and the more dendrite‐resistant microstructures should drastically improve the quality of the final product, making it commercially more attractive. More broadly, carbide‐based approaches should enable optimum microstructures in a range of other oxide and phosphate electrolytes, since many are fabricated using similar co‐mill and then sinter in air routes.^[^ ^98^, ^99^, ^100^ ^]^ Any increase in cost due to the precursors will likewise be offset by the reduced processing temperature and by the overall improved electrolyte performance.

## 3 Conclusion

This is the first report of reactive carbide precursor‐based solid‐state synthesis of NASICON‐type solid‐state electrolyte (SSE), focusing on NZSP (Na_1+x_Zr_2_Si_x_P_3‐x_O_12_). Exothermic decomposition of ZrC and SiC in air homogenizes the microstructure and yields 98% compact density after conventional sintering at 1200 °C. Analysis reveals significant differences in volume fraction and morphology of secondary phases (zirconia, glassy phosphate) for Carb‐NZSP, versus baseline NZSP fabricated from oxide precursors. The Carb‐NZSP is also significantly denser than the baseline. Carb‐NZSP exhibits a zirconia volume fraction of 0.2% ± 0.3%, whereas the baseline is at 3% ± 1%. The glassy phase is agglomerated, while for baseline, it forms a percolated path around the NZSP grains; 50% of the total glass phase accumulates in individual sections larger than 0.48 µm^2^ compared to 0.10 µm^2^ for the baseline. Electrochemical testing demonstrates how microstructure is critical for the suppression of dendrite growth. Carb‐NZSP demonstrates stable Na electrodeposition/dissolution for over 1000 cycles (corresponding to 2000 h) at a constant current density of 0.1 mA cm^−^^2^ and a capacity of 0.1 mAh cm^−^^2^. Critical current density (CCD) is 3.1 ± 0.8 mA cm^−^^2^ at 0.1 mAh cm^−^^2^ versus 1.0 ± 0.7 mA cm^−^^2^ for baseline. Post‐mortem analysis demonstrates 2D sheets of sodium metal dendrites that appear to propagate around the electrolyte grains, through regions that should be rich in pores and in the glassy phase. For Carb‐NZSP, the isolated (vs finely dispersed) pockets of glassy phase and its higher density make dendrite propagation more difficult versus for the baseline. Phase field modelling demonstrates the deleterious nature of the glassy phase on mechanical resistance to dendrite growth. It shows that the presence of finely dispersed glassy phase around the grains of NZSP facilitates dendrite growth and eventual short circuiting. Minimizing this interconnectedness slows dendrite propagation, explaining the measured improvement in the electrochemical stability for Carb‐NZSP.

## Conflict of Interest

The authors declare no conflict of interest.

## Supporting information



Supporting Information

## Data Availability

The data that support the findings of this study are available from the corresponding author upon reasonable request.
