# Supplementary material for: Reactive Carbide‐Based Synthesis and Microstructure of NASICON Sodium Metal All Solid‐State Electrolyte
Source: Adv Mater. 2025 Nov 5;38(16):e12961. doi: 10.1002/adma.202512961 (PMC12994332; doi:10.1002/adma.202512961)
Supplement: Supplementary file 1 — Supporting Information [file ADMA-38-e12961-s001.docx]

# Supporting Information: Reactive Carbide-based Synthesis and Microstructure of NASICON Sodium Metal All Solid-State Electrolyte

Callum J. Campbell^1*^, Scott Monismith^2^, Vikalp Raj^1^, Yixian Wang^1^, Qianqian Yan^3^, Cole D. Fincher^3^_,_ Rohit Raj^1^, Yet-Ming Chiang^3^, John Watt^4^, Josefine D. McBrayer^2^, David Mitlin^1*^

*^1^**Materials Science and Engineering Program & Texas Materials Institute (TMI), The University of Texas at Austin, Austin, TX 78712, United States*

*^2^Power Sources Technology Group, Sandia National Laboratory, Albuquerque, New Mexico, United States*

*^3^* *Department of Materials Science & Engineering, Massachusetts Institute of Technology, Cambridge, MA, 02139, United States*

*^4^Center for Integrated Nanotechnologies, Los Alamos National Laboratory, Los Alamos, NM 87545, United States*

*** *callumcampbell@utexas.edu, david.mitlin2@utexas.edu*

### **Experimental**

### **Synthesis**

Carbide based NZSP was synthesized using a conventional solid-state synthesis route. The precursors included anhydrous sodium carbonate (Na_2_CO_3_, Alfa, 99.5%), ammonium biphosphate (NH_4_H_2_PO_4_, Acros, 99.9%), zirconium carbide (ZrC, Thermo Scientific, 99.5%), and silicon carbide (SiC, Thermo Scientific, 600 grit). Stoichiometric quantities of the precursors were weighed to produce between 4 g and 15 g of NZSP, incorporating a 25% excess of Na_2_CO_3_ and 10% excess of NH_4_H_2_PO_4_. The powders were mixed in a granite mortar and pestle for 10 minutes and annealed in an alumina crucible at 1050 °C for 12 hours to yield annealed powder. The annealed powders were lightly crushed in a granite mortar and then wet-milled using a planetary ball mill with ethanol as the solvent at 350 RPM for 20 hours. The ball-to-powder-to-solvent ratio was maintained at 4:2:1 by weight during milling. After drying, the powders were pressed into green compacts (600 mg, 12.7 mm diameter) using a steel die under an approximate load of 2,000 kg. The green compacts were sintered in alumina crucibles with a thin layer of "mother powder" at the base to minimize contamination. Sintering was conducted in a box furnace under ambient conditions using a multistep heating protocol: 1175 °C for 2 hours, 1150 °C for 12 hours, and 900 °C for 1 hour, with a ramp rate of 10 °C/min for both heating and cooling. Following sintering, the compacts were dry polished to a 4000-grit finish and subjected to a heat treatment at 1100 °C for 30 minutes in air. This additional heat treatment is known to improve the interfacial impedance with Na metal^[1]^.

For the baseline oxide-precursor based NZSP, a well-established solid-state synthesis route was followed. Apart from the selection of precursors, the process mirrors the carbide-based route. The precursors included anhydrous sodium carbonate (Na_2_CO_3_, Alfa, 99.5%), ammonium biphosphate (NH_4_H_2_PO_4_, Acros, 99.9%), zirconium oxide (ZrO_2_, Alfa Aesar, 99.7%), and silicon dioxide (SiO_2_, Thermo Scientific, 10-20nm, 99.5%). Stoichiometric quantities were weighed to produce approximately 8 g of NZSP, with 25% excess Na_2_CO_3_ and 10% excess NH_4_H_2_PO_4_. The precursor mixture was wet milled in a planetary ball mill with ethanol at 350 RPM for 20 hours and then dried. The dried powder was annealed in a box furnace at 1150 °C for 12 hours, followed by a second milling step under identical conditions for 20 hours. The milled powder was pressed into green compacts (600 mg, 12 mm diameter) using a steel die under an approximate load of 2,000 kg. The green compacts were sintered in alumina crucibles with a thin layer of "mother powder" at the base, employing a multistep heating protocol: 1240 °C for 2 hours, 1200 °C for 12 hours, and 1000 °C for 1 hour, with a ramp rate of 10 °C/min. The sintered compacts were dry polished to a 4000-grit finish and heat-treated at 1100 °C for 30 minutes in air to enhance interfacial impedance with Na metal. The proposed net chemical reactions during the procedure are presented in Equation 1 and Equation 2 for Carb-NZSP based and oxide based NZSP, respectively.

$$\begin{aligned} 1.5{Na}_{2}CO_{3} + 2SiC+ 2ZrC+ NH_{4}H_{2}PO_{4} +8O_{2} \#(1) \\ \to{Na}_{3}{Zr}_{2}{Si}_{2}PO_{12} + 5.5CO_{2} + 1.5H_{2}O+{NH}_{3} \# \end{aligned}$$

$$\begin{aligned} 1.5{Na}_{2}CO_{3} + 2SiO_{2}+ 2ZrO_{2}+ NH_{4}H_{2}PO_{4} \#(2) \\ \to{Na}_{3}{Zr}_{2}{Si}_{2}PO_{12} + 1.5CO_{2} + 1.5H_{2}O+NH_{3}\# \end{aligned}$$

### **Analytical Characterization**

The relative density of the compacts was measured through Archimedes principle, using a custom-built setup and a weighing balance (Sartorius ED124). Powder X-ray diffraction was performed on crushed pellets on a Rigaku-600 benchtop diffractometer using a Cu K-α X-ray source with monochromator. PXRD spectra were collected between a 2θ angle of 10° and 50° at a scan rate of 0.3 ° min^-1^. Rietveld refinement was performed using Fullprof software. For the Carb-NZSP and NZSP fits, the χ^2^ values were 7.5 and 7.7, respectively. Particle size analysis of the annealed but not sintered powder precursors was performed via sonicating the powders in acetone, drop casting onto copper foil attached to SEM stubs by carbon tape, then dried in vacuum without heat. SEM images were analyzed using ImageJ-1.54g by the longest and shortest cord measurements of 50 selected particles.

Vickers hardness testing was performed on sintered and heat treated NZSP and Carb-NZSP pellets. All micro-indentations were performed inside an Ar glovebox (H_2_O, O_2_ < 1 ppm; 25 °C) using a Phase II Vickers microhardness tester (Model 900-390) equipped with a 136° diamond pyramid indenter and an integrated optical microscope (50× objective). Fracture was observed only once in a single trial with Carb-NZSP, and so quantification of fracture toughness is not attempted.

SEM imaging of fracture surfaces was performed using a Scios 2 Dual Beam SEM-FIB system and an Apreo 2C LoVac system. Energy Dispersive X-ray Spectroscopy (EDXS) analysis was performed using a Bruker XFlash 7. The SEM images were taken using either an Everhart-Thornley Detector (ETD) or backscatter detector at a voltage range of 2-20 kV with a working distance of 7-10 mm. SEM-EDXS was performed at a voltage of 20 kV with a working distance of 7-10 mm. Quantitative stereology analysis was performed using the software ImageJ.

For phase quantification, SEM-FIB imaging was performed in a Scios Dual-Beam FIB/SEM. Sintered compacts of Carb-NZSP and baseline NZSP were fractured and dry polished to 7000 grit, before mounting the fracture sections horizontally. The largest three flaws observed in the polished fracture surface for both NZSP and Carb-NZSP were imaged at low magnification under the SEM at 10 keV and 0.1 nA with secondary electron images presented in **Figure S4.** To evaluate the variation in microstructural features throughout the bulk, various regions were selected from near the pellet center, face, and edge, as shown in **Figures S7.** Regions of interest for quantitative analysis were selected from within the bulk, near the pellet center. Within the SEM-FIB chamber, a platinum protective layer was deposited over regions of interest via a Gas Injection System (GIS) and cured by the Ga^+^-ion beam. Trenches were cut into the exposed surfaces with Ga^+^-ion currents utilizing staircase patterning between 7 nA – 15 nA, followed by cleaning cross sections between 1-3 nA to reduce curtaining and clean the interface of redeposited material. Exposed and cleaned FIB sections were imaged with an in-column backscatter detector at 2 keV, 50 pA, with beam deceleration using Opti-Tilt functionality. ImageJ-1.54g processing software was used to process, threshold, and analyze particles. After nearest-neighbor smoothing with a Gaussian filter, shadows were removed from images using the techniques outlined in Supplementary **Figure S18**. Automated particle analysis was carried out, which provided measurements of particle area from pixel count, and perimeter measurement with edge pixels as one unit and corner pixels as $\sqrt{2}$ units. The total ‘glassy phase’ and ‘porosity’ (dark contrast) and ‘zirconia phase’ (bright contrast) were quantified by area fraction in each image, manually reviewed by the authors, and the remaining area attributed to crystalline NZSP. Assuming uniform, isotropic, random sampling of the cross sections in the bulk, the area fractions measured in the cross sections are identical to the volume fractions in the 3D structure^[2]^. To quantify the phase distributions, particle analysis was conducted on zirconia particles, as well as individually identified glass sections. Aside from grain size, the stereological analysis relating size and shape distributions of particles in 2D cross section to the 3D structure are heavily influenced by assumptions about the microstructure. We instead focus the analysis of size distribution on direct metrics (such as cross section area) of distributions in the 2D cross sections and make direct comparisons to the baseline samples.

Cryogenic STEM-EELS was performed in a Titan ETM with 300 keV accelerating voltage, using a dual range near-simultaneous high loss and low loss data collection on a BioContinuum HD/K3-IS Electron Energy-Loss Spectrometer (EELS) detector. Sample lamellae were prepared by cryogenic SEM-FIB using a Scios 2 with a Leica cryogenic stage installed. Sample lamellae were cut, lifted out, and attached to the copper TEM grid at room temperature before cooling to -150 °C for sample thinning to minimize beam damage. Moderate water ice contamination is observable in cryo-STEM images but does not preclude structural observations or elemental mapping. Elemental quantification was performed using Gatan Digital Micrograph software with EELS Quantification. Spectra were fit with a Hartree-Slater model without plural scattering corrections. Spectra of specific regions within the EELS maps were normalized by division of the maximum intensity value within the spectrum range, as shown in **Figure S6.** The ‘Whole Area’ in **Figure S6(d)** refers to the survey region presented in **Figure 2(d).**

Thermogravimetric analysis and differential scanning calorimetry (TGA-DSC) were performed on a Netzsch STA 449 F1 on the as-milled precursors prior to annealing for both Carb-NZSP and baseline NZSP. Additionally, TGA-DSC was conducted on the ZiC and SiC reagent powders. For all samples, small quantities of powder (5–15 mg) were loaded into alumina (Al_2_O_3_) crucibles and heated from room temperature to 1300 °C at a rate of 20 °C min⁻¹ under a gas flow comprising 150 mL min⁻¹ of air and 20 mL min⁻¹ of protective nitrogen. Correction measurements were performed using empty crucibles under the same conditions prior to sample loading, and the resulting baseline was subtracted from the sample measurement after the measurement. Calculation of reaction enthalpies was performed using values from the Materials Project Database for Reaction 1 and Reaction 2 **(Table S10)**.

### **Electroanalytical Characterization**

Na-ion blocking cells were utilized to determine the ionic conductivities and activation energies of both baseline NZSP and Carb-NZSP solid electrolytes (SEs). For the blocking cells, a 100 nm tungsten (W) layer was sputtered onto both sides of the polished SE, via RF magnetron sputtering using an AJA Orion ATC instrument. The deposition was performed at a rate of 0.10 nm s^-1^ while maintaining a deposition pressure of 3 mTorr. The cells were placed in a PEEK cell with indium contacts on each side to ensure good interfacial contact. Potentiostatic electrochemical impedance spectroscopy (EIS) was performed using a Gamry 600+ Potentiostat/Galvanostat/FRA, applying a 10 mV signal over a frequency range of 5 MHz to 1 Hz. Curve fitting was conducted using ZSIMPWIN software. Models were fit with an R(RQ)(RQ)Q equivalent circuit, as shown in **Figure S12(d)**. The solid electrolyte resistance is attributed to the sum of the series resistor, R_1_, and the high frequency (lowest capacitance) resistance R_2_.

$$R_{SE}=R_{1}+ R_{2}$$

To evaluate the sodium electrodeposition/dissolution behavior of the synthesized compacts, symmetric cells were assembled in a Ni|Na|NZSP|Na|Ni configuration, employing sodium metal as the anode and nickel as the current collector. Sodium metal was first rolled into thin sheets, from which 6 mm diameter discs were punched. These sodium discs were pressed onto either side of the SE compact, followed by 8 mm diameter nickel current collectors. The entire cell was momentarily subjected to a uniaxial pressure of approximately 10 MPa, then heated at 150 °C for 6 hours. PEEK cells with titanium rods were assembled in an argon filled glovebox with < 0.1 ppm O_2_ and < 0.1 ppm H_2_O, then sealed with silicone vacuum grease. Sealed PEEK cells were tested outside the glovebox. Interfacial impedance measurements were made using the same EIS protocol described above. Fitting was performed as before, with an R(RQ)(RQ)Q circuit element, with the low frequency (largest capacitance) R_3_ attributed to interfacial resistance. Electrochemical cycling was carried out in several configurations. All galvanostatic cycling experiments were conducted using a PARSTAT PMC 200 Potentiostat (Princeton Applied Research) with VersaStudio 2.61.3 software. For extended cycling, cells were run galvanostatically under approximately 5 MPa of pressure at a current density of either 0.1 mAh cm^-2^, 0.5 mA cm^-2^, or 1.0 mA cm^-2^ for 1 hour half cycles to give capacities of 0.1 mAh cm^-2^, 0.5 mAh cm^-2^, or 1.0 mAh cm^-2^, respectively. Critical current density (CCD) measurements were performed using a constant capacity cycling protocol, beginning at a current density of 0.2 mA cm⁻² and incrementing by 0.2 mA cm^-2^ with each cycle at a capacity of 0.1 mAh cm^-2^. To prepare baseline NZSP cells for dendrite investigation, cycling was performed at 0.1 mA cm^-2^ and 0.1 mAh cm^-2^ for five cycles before increasing the current to 0.5 mA cm^-2^ until short circuit.

To evaluate the impact of cycling at elevated temperatures, half cells of Carb-NZSP were assembled with 8mm Ni current collectors, 6mm Na foil, and assembled in coin cells before going through extended cycling at 55°C. Low current cycling was performed at 0.1 mA cm^-2^ for 0.1 mAh cm^-2^ for 5 cycles followed by 0.5 mA cm^-2^ and 0.5 mAh cm^-2^  continuous cycling. High current cycling was performed at 0.5 mA cm^-2^ and 0.5 mAh cm^-2^ for 5 cycles, followed by 3.0 mA cm^-2^ and 3.0 mAh cm^-2^ with polarization observed around 15.8 hours. The high and low current cycling potential profiles are presented in **Figure S19(a) – (b)**, respectively.

For the asymmetric configuration, polished and heat treated NZSP pellets were coated with 10 nm of Te on one side via RF magnetron sputtering using an AJA Orion ATC instrument. The deposition was performed at a rate of 0.22 nm s^-1^ while maintaining a deposition pressure of 60 mTorr. The remaining surface was interfaced with sodium metal, and both ends with a nickel current collector to make Ni|Na|NZSP|Te|Ni asymmetric cells. In order to consistently cycle appreciable capacity, asymmetric cells were cycled at an elevated temperature of 55°C. In asymmetric cells, sodium was plated onto the Ni|Te current collector at a current density of 0.1 mA cm^-^² for 3600 seconds, corresponding to a capacity of 0.1 mAh cm^-^². Stripping was performed at the same current density of 0.1 mAh cm^-^² with a cutoff voltage and capacity of 1 V and 0.1 mAh cm^-^², respectively. Asymmetric Carb-NZSP were prepared similarly, with cycling 55°C at higher current capacities of 0.5 mA cm^-2^ and 0.5 mAh cm^-2^ for 5 cycles, then at 1.0 mA cm^-2^ and 1.0 mA cm^-2^ until short circuit.

All cycled cells were disassembled in an argon filled glovebox with < 1 ppm H_2_O and < 3 ppm O_2_. Ni current collectors were mechanically removed, and samples were mounted via copper or carbon tape. A Lecia VCT-500 cryogenic/air sensitive shuttle was used to transfer samples into the cryogenic SEM-FIB. All post-mortem SEM-FIB imaging and milling was performed on a liquid nitrogen cooled stage at cryogenic temperatures (-150°C) to minimize beam damage and chemical reaction between sodium metal and gallium from the ion beam. A platinum protective layer was deposited over regions of interest via a Gas Injection System (GIS) and cured by the Ga^+^-ion beam. Trenches were cut and cleaned with a 30 keV Ga^+^ ion beam at 0.3-15 nA. For analysis of the NZSP interface, trenches were milled through the Na metal foil (50-70 μm, 15 nA Ga^+^ beam, 1-3 nA cleaning) until the interface was observed. Secondary and backscatter SEM images were taken with in-column detectors with a 2 keV landing voltage, 50 pA approximate beam current, 52° stage tilt, with beam deceleration via the Opti-Tilt functionality.

Full cells utilizing heat treated Carb-NZSP were assembled by the interfacing Na metal to one side of the electrolyte as in the symmetric cell configuration. Commercial NVP cathode particles were hand mixed into a slurry with a 7:2:1 mass ration of active material: SuperP conductive carbon: PVDF binder in an NMP solvent. Castings were dried on a hot plate in air at 80 C overnight before drying in a vacuum at 80C overnight. Cathode active material mass loading was 6.2 mg cm^-2^. Cells were assembled in a Ar glovebox with H_2_O and O_2_ ppm < 0.01. 8mm diameter cathodes were infiltrated with a small quantity of liquid electrolyte (LE); 50 μL of 1M NaPF_6_ in Diglyme and covered with a single layer Celgard 2400 separator to aid contact with Carb-NZSP. The full Ni|NVP|LE|Carb-NZSP|Na|Ni stack was placed in a coin cell and cycled at 0.1C rate, or 74 μA cm^-2^ for 645 hours. Full cell voltage profiles are shown in **Figure S19(c)** and cycle capacities and coulombic efficiencies are shown in **Figure S19(d)**. The cell demonstrates stable cycling behavior.

**Simulation Methods**

A more complete description of this framework can be found in a prior work^[3]^, but the system of equations is shown below:

$\begin{aligned} F=\int f_{chem}\left( \xi\right)+f_{grad}\left( \nabla\xi,\theta\right)+f_{mech}\left( u_{i},\phi\right)dV, \#\left( 1 \right) \end{aligned}$

$$\begin{aligned} \nabla\cdot\left( \sigma^{eff}\nabla\Psi\right)=nc_{s}F\frac{\partial\xi}{\partial t}, \#\left( 2 \right) \end{aligned}$$

$$\begin{aligned} \frac{\partial\tilde{c}}{\partial t}=\nabla\cdot\left[ D_{\mathrm{Na}^{+}}^{\mathrm{eff}}\nabla\tilde{c}+D_{\mathrm{Na}^{+}}^{\mathrm{eff}}\frac{\tilde{c}\mathrm{zF}}{\mathrm{RT}}\nabla\Phi\right] , \#\left( 3 \right) \end{aligned}$$

$$\begin{aligned} \nabla\cdot\left( \boldsymbol{\sigma}^{\boldsymbol{0}}\boldsymbol{+}\boldsymbol{\sigma}^{\boldsymbol{\eta}} \right)=0, \#\left( 4 \right) \end{aligned}$$

$$\begin{aligned} -l^{2}\nabla^{2}\Phi+\Phi+\frac{2l}{G_{c}} \left( 1-\Phi\right)H, \#\left( 5 \right) \end{aligned}$$

$$\begin{aligned} \frac{\partial\xi}{\partial t}=g\left[ L_{\xi}\left[ \frac{\partial f_{chem}}{\partial\xi}+\frac{\partial f_{mech}}{\partial\xi}-\kappa_{\xi}^{0}\left[ 1+\delta\cos\left( \omega\theta\right) \right]\nabla^{2}\xi\right]+i_{BV} \right]. \#\left( 6 \right) \end{aligned}$$

Equation 1 is the Ginzburg-Landau free energy functional where f_chem_ is the chemical free energy of the system, f_grad_ is the gradient free energy (related to surface energies) and f_mech_ is the mechanical free energy. Equation 2 is the Poisson equation, where σ^eff^ is the effective electrical conductivity, Ψ is the electric potential, n is the number of electrons exchanged in the reaction, c_s_ is the site density of ions in the Na metal, F is the Faraday constant, and $\frac{\partial\xi}{\partial t}$ is the derivative of the Na metal order parameter with respect to time (the reaction rate). In combination with Eq. 2, Eq. 3 completes the Nernst-Poisson-Planck model governing current and species conservation; $D_{Na^{+}}^{eff}$ is the effective Na^+^-ion diffusion coefficient,  $\tilde{c}$ is the ion concentration normalized by the equilibrium value in the material, z is the electron charge, R is the gas constant, and T is the temperature. Equation 4 & 5 are the two equations that govern mechanical equilibrium and the growth of the crack phase-field variable respectively. The stress σ^η^ is the hydrostatic pressure that develops inside the growing Na filament, while σ^0^ is the stress tensor that arises in response to this pressure. The evolution of the crack phase-field variable, Φ in Eq. 5 is dictated by *l*, the internal length-scale parameter and H, the strain energy history field that reflects the maximum tensile strain energy over the full time history of the simulation. A more thorough accounting of this type of fracture phase-field model may be found in the phase-field fracture literature^[4,5]^. Lastly, equation 6 describes the evolution of the Na order parameter, where L_η_ is the interfacial mobility, i_BV_ is the reaction rate calculated from a Butler-Volmer model of electrodeposition, $\kappa_{\xi}^{0}$ is the gradient energy coefficient, and ω, θ, and δ are the mode, angle, and strength of the anisotropic gradient energy. Critically, this equation is prefaced by g, a switching function that interpolates between damaged and undamaged material (i.e. where Φ = 0, g = 0 and where Φ = 1, g = 1). This enforces that the Na filaments may only grow where there are pre-existing cracks to fill. To solve the system of Equations 1-6, the Sandia National Lab’s phase-field code, MEsoscale Multiphysics PHase-fIeld Simulator (MEMPHIS) was used^[6,7]^.

***
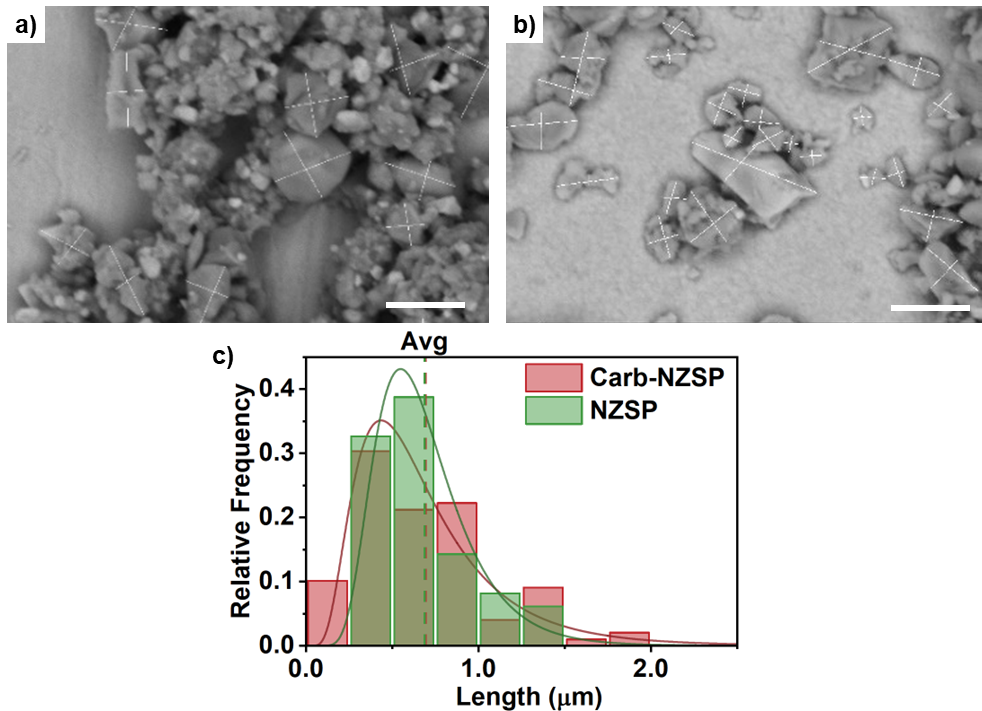

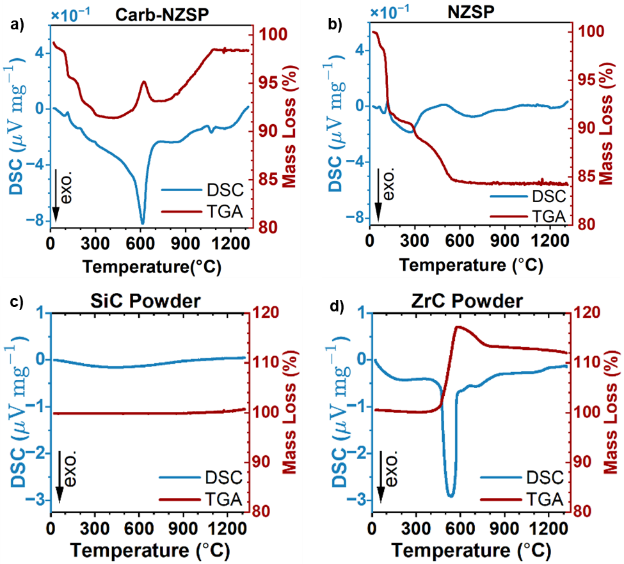
***

**Figure S1**. SEM images of dispersed green powders of (a) Carb-NZSP and (b) oxide based NZSP with scale bar of 1 μm. (c) Particle size distribution green powders.

**Figure S2.** A plot of TGA-DSC for (a) Carb-NZSP precursor mixture, (b) NZSP precursor mixture, (c) SiC powder, and (d) ZrC powder.

**Figure S3.** Rietveld refinement results for a) Carb-NZSP and b) Oxide-based NZSP.


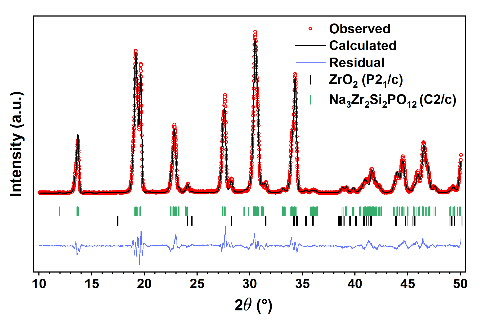

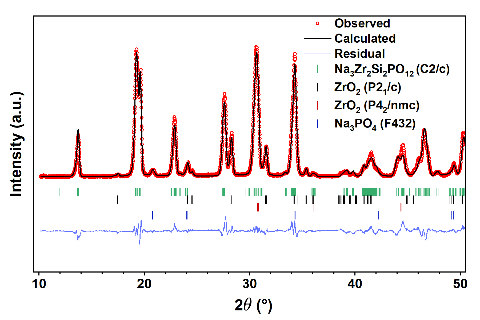


**a)**

**b)**

**Table S1.** Rietveld refinement lattice parameters for a) Carb-NZSP and b) Oxide-based NZSP.

**Table S1.** Rietveld refinement lattice parameters for a) Carb-NZSP and b) Oxide-based NZSP.

| Sample | a [Å] | b [Å] | c [Å] | γ [°] | V [Å^3^] |
| --- | --- | --- | --- | --- | --- |
| Carbide (a) | 15.67(2) | 9.069(9) | 9.218(0) | 123.88(6) | 1087.(8) |
| Oxide (b) | 15.68(8) | 9.07(4) | 9.19(6) | 124.12(6) | 1083.(7) |

| Carb-NZSP | x | y | z | B | occ. |
| --- | --- | --- | --- | --- | --- |
| P1 | 0.000 | 0.080 | 0.250 | 1.000 | 0.121 |
| Na2 | 0.250 | 0.250 | 0.500 | 1.000 | 0.216 |
| Na3 | 0.500 | 0.876 | 0.250 | 1.000 | 0.431 |
| P4 | 0.371 | 0.097 | 0.276 | 1.000 | 0.387 |
| Zr5 | 0.105 | 0.246 | 0.053 | 1.262 | 0.953 |
| Si6 | 0.371 | 0.097 | 0.276 | 1.000 | 0.662 |
| Na7 | 0.827 | 0.077 | 0.824 | 1.000 | 0.795 |
| O8 | 0.083 | 0.156 | 0.233 | 1.000 | 1.000 |
| O9 | 0.455 | 0.191 | 0.450 | 1.000 | 1.000 |
| O10 | 0.163 | 0.431 | 0.289 | 1.000 | 1.000 |
| Si11 | 0.000 | 0.080 | 0.250 | 1.000 | 0.339 |
| O12 | 0.378 | 0.121 | 0.103 | 1.000 | 1.000 |
| O13 | 0.442 | 0.479 | 0.114 | 1.000 | 1.000 |
| O14 | 0.254 | 0.198 | 0.246 | 1.000 | 1.000 |

**Table S2.** Rietveld refinement atomic positions and occupancies for Carb-NZSP in the C2/c space group.

| NZSP | x | y | z | B | occ. |
| --- | --- | --- | --- | --- | --- |
| P1 | 0.000 | 0.051 | 0.250 | 1.000 | 0.143 |
| Na2 | 0.250 | 0.250 | 0.500 | 1.000 | 0.388 |
| Na3 | 0.500 | 0.885 | 0.250 | 1.000 | 0.488 |
| P4 | 0.348 | 0.121 | 0.250 | 1.000 | 0.364 |
| Zr5 | 0.104 | 0.251 | 0.055 | 1.000 | 0.996 |
| Si6 | 0.348 | 0.121 | 0.250 | 1.000 | 0.701 |
| Na7 | 0.829 | 0.092 | 0.825 | 1.000 | 0.821 |
| O8 | 0.086 | 0.132 | 0.248 | 1.000 | 1.000 |
| O9 | 0.440 | 0.184 | 0.435 | 1.000 | 1.000 |
| O10 | 0.145 | 0.436 | 0.225 | 1.000 | 1.000 |
| Si11 | 0.000 | 0.051 | 0.250 | 1.000 | 0.335 |
| O12 | 0.375 | 0.130 | 0.109 | 1.000 | 1.000 |
| O13 | 0.434 | 0.448 | 0.094 | 1.000 | 1.000 |
| O14 | 0.243 | 0.187 | 0.195 | 1.000 | 1.000 |

**Table S3.** Rietveld refinement atomic positions and occupancies for baseline NZSP in the C2/c space group.

***
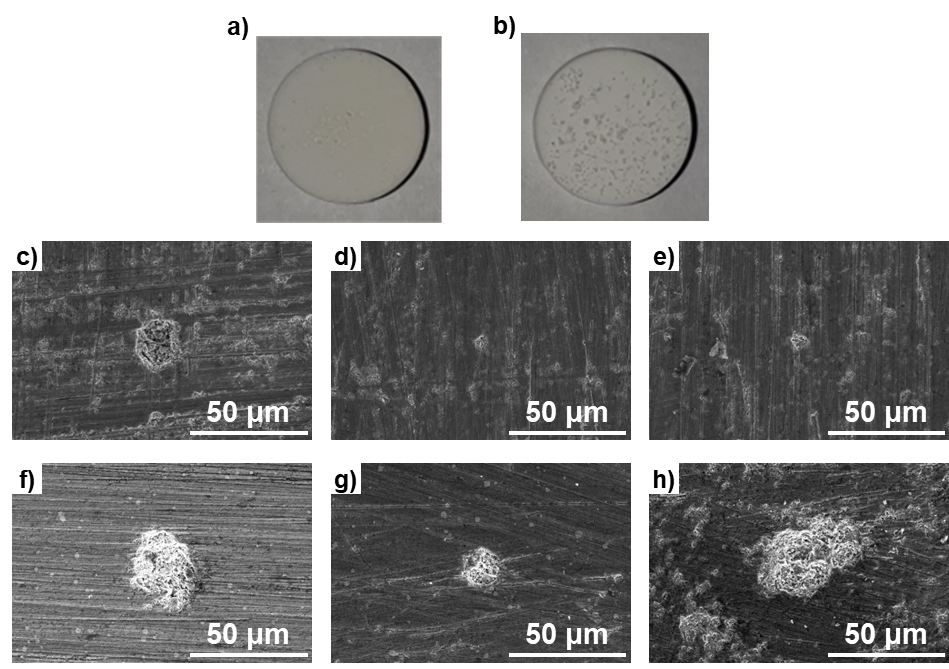
***

**Figure S4:** Digital photographs of a) Carb-NZSP and b) NZSP bottom sintered surfaces after rough polishing. c-h) Secondary electron image of flaws observed on polished fracture surfaces within the bulk of the Carb-NZSP and NZSP solid electrolyte compacts. c-e) Largest three flaws observed in the polished fracture surface of a Carb-NZSP pellet. f-h) largest three flaws observed in the NZSP polished fracture surface.

### **
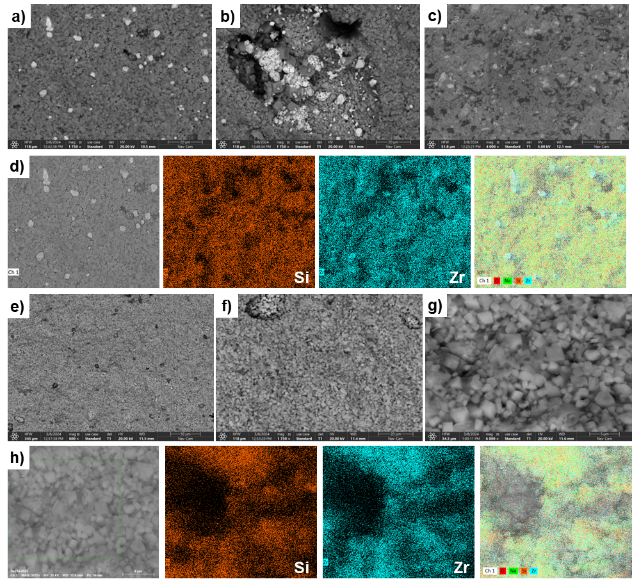
**

**Figure S5.** Cross-sectional SEM and corresponding EDX images for fractured surfaces of Oxide based NZSP (a-d) and Carb-NZSP (e-h).

**Figure S6:** Normalized spectra for various regions of the cryo-STEM EELS maps shown in Figure 2. The regions of interest quantified for direct comparison of a) the Carb-NZSP crystallite and b) the Na-P rich glass phase, and c) the zirconia grains. d) Normalized spectra of the whole survey region, the NZSP crystallite as shown in a) and the glass region as shown in b), and the zirconia region shown in c). Inset shows the Na K edge at 1072 eV is observed in the NZSP and glass, but not in the zirconia region.


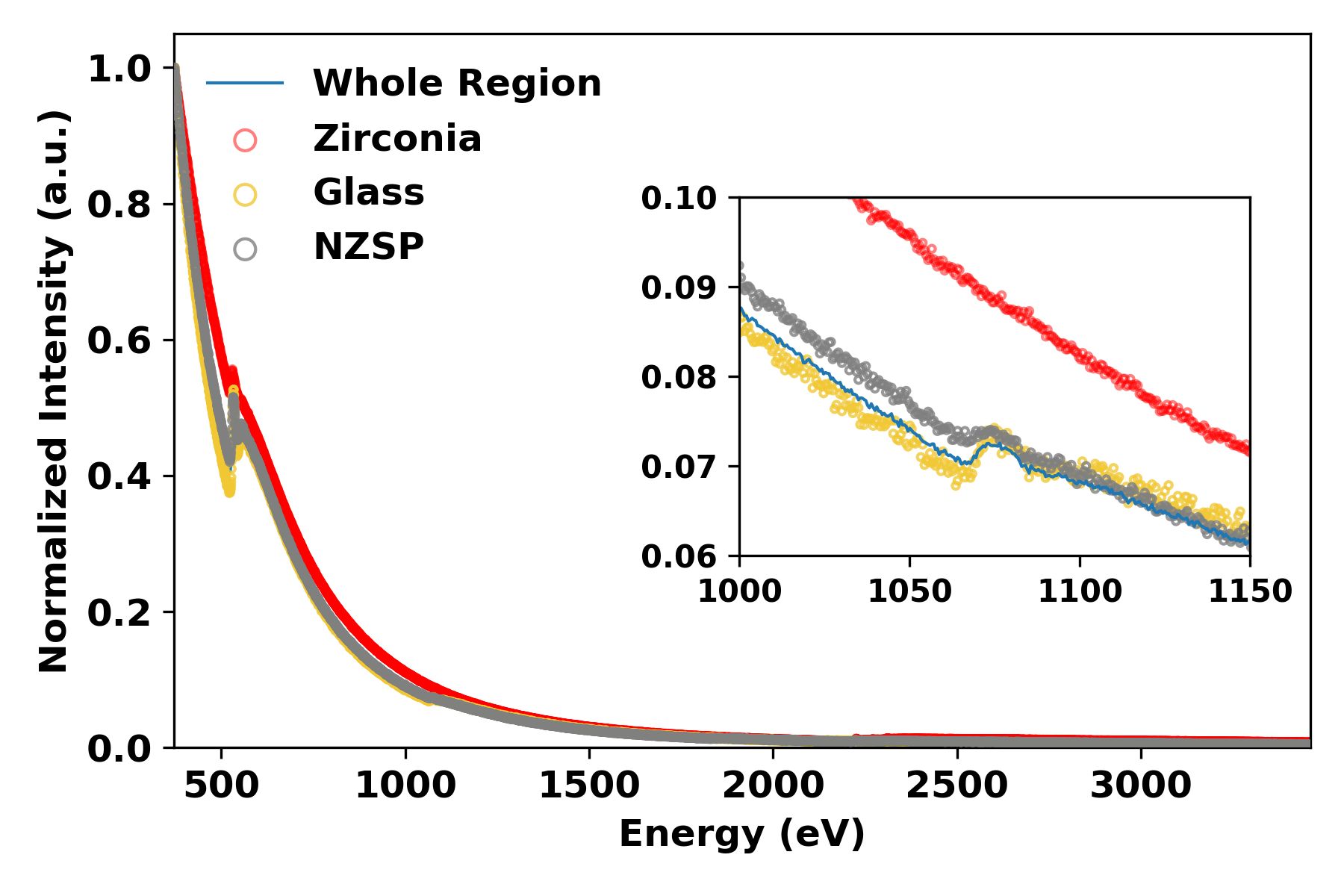


**a)**

**b)**


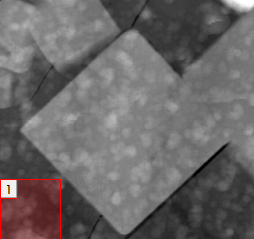

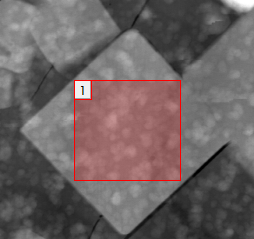

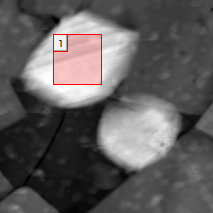


**c)**

**
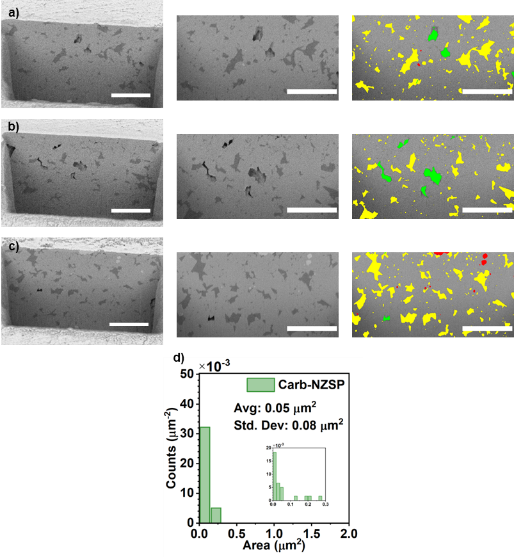

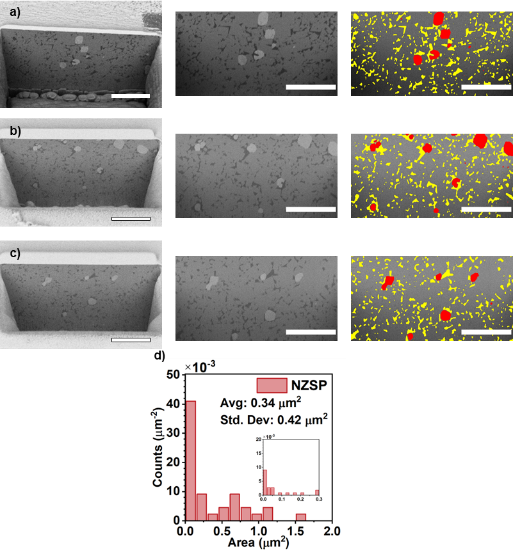
**

**Figure S7.** FIB cross sectional SEM images and corresponding stereology images mapping zirconia particles (in red), glassy phase (yellow) and porosity (green) for Carb-NZSP over multiple areas (a), (b), (c). d) Histogram plot showing the zirconia particle cross sectional area distribution. Scale bars are 5 μm.

**Figure S8.** FIB cross sectional SEM images and corresponding stereology images mapping zirconia particles (red), glassy phase (yellow) and porosity (green) for oxide based NZSP over multiple areas (a), (b), (c). d) Histogram plot showing the zirconia particle cross sectional area distribution. Scale bars are 5 μm.

|  | Image | Total Area [μm^2^] | Pores [%] | Glass [%] | Zirconia [%] | NZSP [%] |
| --- | --- | --- | --- | --- | --- | --- |
| Carb-NZSP | a | 199.61 | 0.76 | 8.97 | 0.04 | 90.23 |
|  | b | 203.01 | 1.47 | 7.03 | 0.03 | 91.48 |
|  | c | 191.45 | 0.32 | 11.32 | 0.52 | 87.84 |
|  | Average: | 200.8 | 0.85 | 9.10 | 0.19 | 89.85 |
| Oxide NZSP | a | 142.64 | 0.00 | 12.93 | 2.54 | 84.53 |
|  | b | 137.96 | 0.00 | 12.23 | 3.91 | 83.87 |
|  | c | 161.09 | 0.00 | 9.91 | 1.86 | 88.23 |
|  | Average: | 147.2 | 0.00 | 11.69 | 2.77 | 85.54 |

**
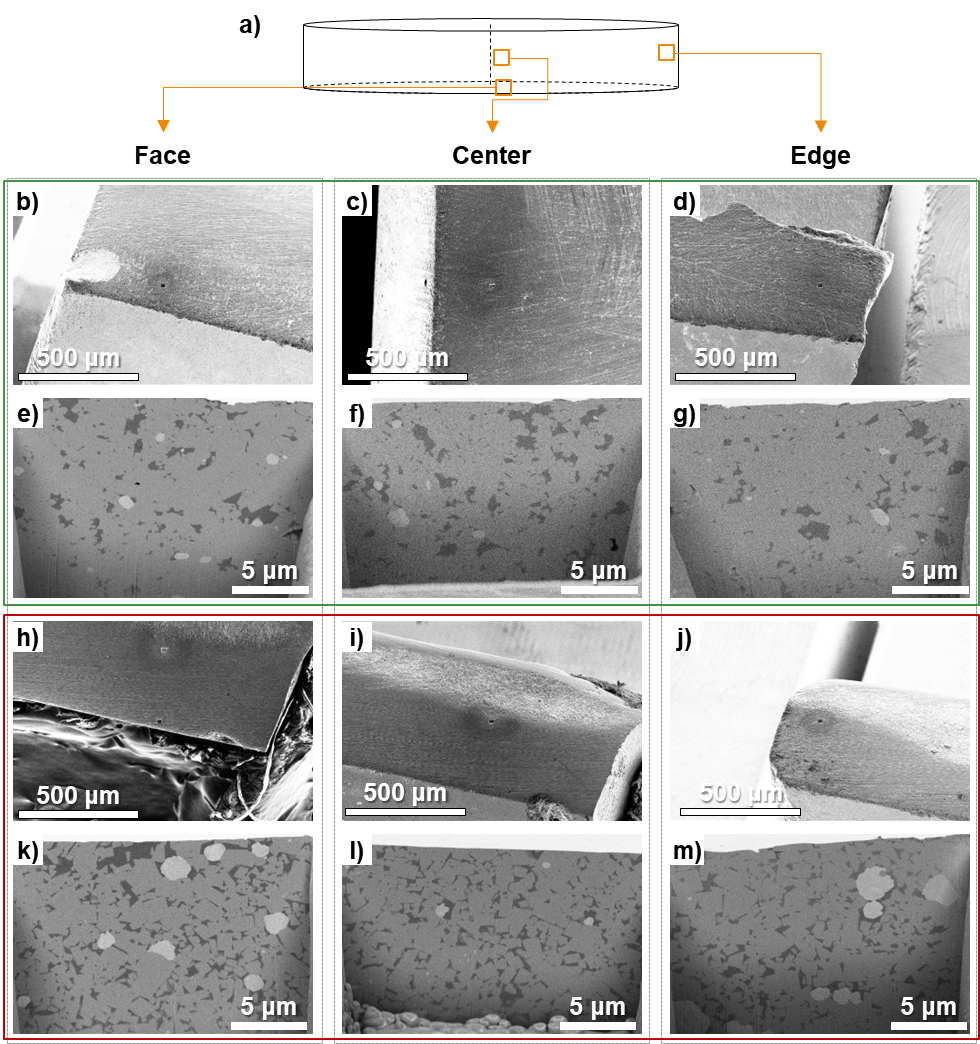

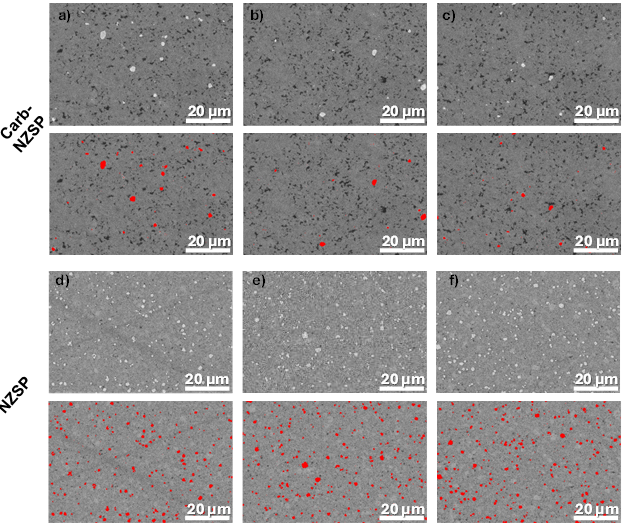
**

**Table S4.** Quantification of the FIB cross section phase composition. Total sample area and area fractions of FIB sections in Figures SI 6 and SI 7.

**Figure S9:** Comparison of FIB regions throughout the bulk of the pellets for Carb-NZSP (green box) and NZSP (red box). (a) diagram of position of regions selected from the bulk for FIB sectioning, including the center, face, and outside edge of each pellet. (b-d) Carb-NZSP low magnification secondary electron images centered on FIB regions, and (e-g) high magnification backscatter images of the respective cross sections. (h-m) similar analysis for baseline NZSP.

**Figure S10.** Fractured polished cross-sectional SEM images and corresponding stereology maps highlighting zirconia particles (marked in red) for Carb-NZSP (a–c) and oxide-based NZSP (e–g).

|  | Image  Label | Sample size  [μm^2^] | Count | Avg. Size, Ā  [μm^2^] | Median Size  [μm^2^] | Area  [%] |
| --- | --- | --- | --- | --- | --- | --- |
| NZSP | A | 4600 | 324 | 0.54 | 0.34 | 3.78 |
|  | B | 4600 | 295 | 0.53 | 0.37 | 3.38 |
|  | C | 4600 | 260 | 0.52 | 0.33 | 2.92 |
|  |  | 4600 | 879 | 0.53 ± 0.01 | 0.35 | 3.36 |
| Carb-NZSP | E | 4600 | 72 | 0.54 | 0.075 | 0.85 |
|  | F | 4600 | 48 | 0.42 | 0.058 | 0.44 |
|  | G | 4600 | 72 | 0.29 | 0.057 | 0.45 |
|  |  | 4600 | 64 | 0.42 ± 0.13 | 0.06 | 0.58 |

|  | Image | Sample Area  [μm^2^] | Average  Section  Size, Ā [μm^2^] | Median  Size  [μm^2^] | Total Glass  Area  [μm^2^] | Perimeter  [μm] | L_A_  [μm^-1^] |
| --- | --- | --- | --- | --- | --- | --- | --- |
| Carb-NZSP | a | 199.61 | 0.075 | 0.008(5) | 17.91 | 207.26 | 11.57 |
|  | b | 203.01 | 0.072 | 0.010 | 14.27 | 176.157 | 12.35 |
|  | c | 191.45 | 0.079 | 0.007 | 21.67 | 236.378 | 10.91 |
|  | Summary: | 602.24 | **0.076** | 0.009 | 17.95 | 206.0 | **11.6 ± 0.7** |
| NZSP | a | 142.64 | 0.043 | 0.017 | 18.44 | 343.32 | 18.62 |
|  | b | 137.96 | 0.046 | 0.017(5) | 16.86 | 307.11 | 18.21 |
|  | c | 161.09 | 0.040 | 0.019 | 15.97 | 310.92 | 19.47 |
|  | Summary | 441.69 | **0.043** | 0.018 | 17.09 | 320.45 | **18.7 ± 0.6** |

**Table S6.** Quantification of the FIB cross section glass phase distribution.

**Table S5.** Zirconia particle analysis results for polished fracture surfaces.

**
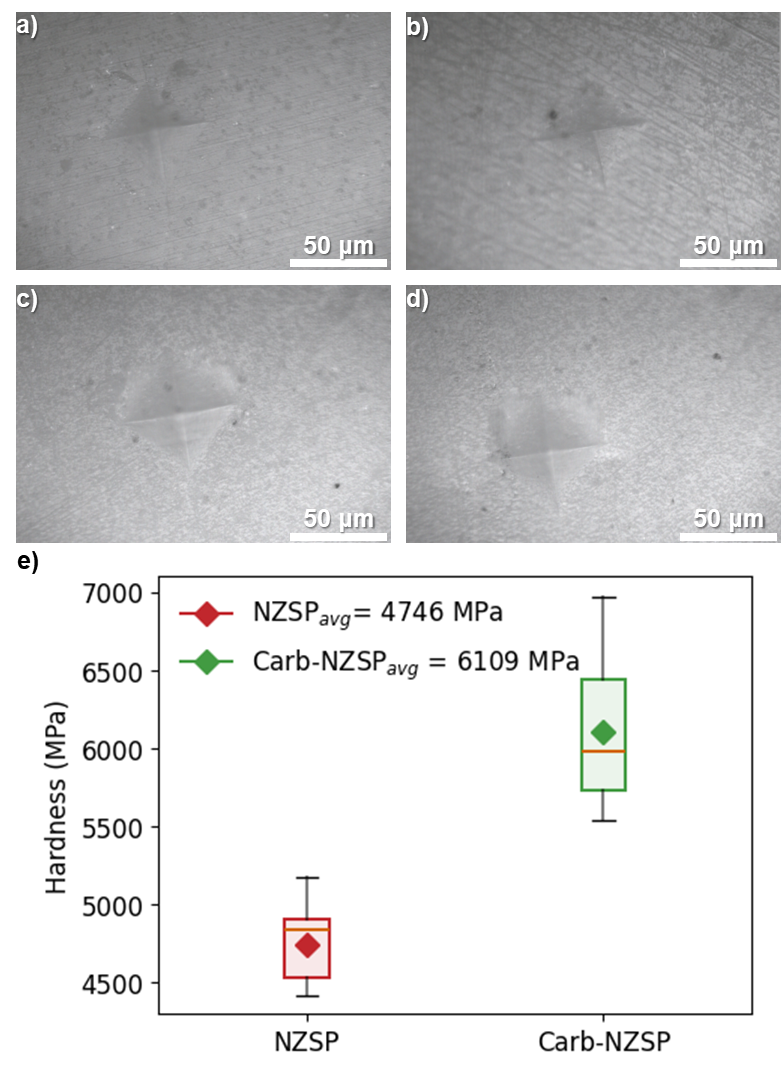
**

**Figure S11.** Optical micrographs of micro-indents from Vickers hardness testing for a-b) NZSP and c-d) Carb-NZSP. e) Hardness testing results for NZSP (red) and Carb-NZSP (green)

**
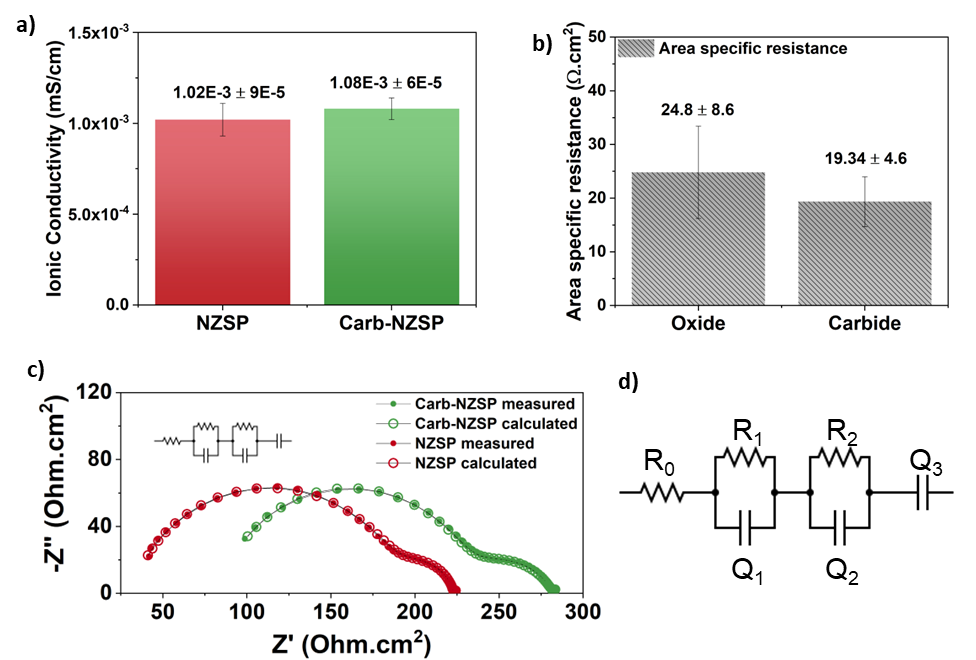
**

**Figure S12**: (a) A bar plot comparing ionic conductivity calculated from EIS of Na-Blocking cell for NZSP and Carb-NZSP. (b) A bar plot comparing area specific resistance calculated from the EIS of symmetric cells for Carb-NZSP and NZSP. (c) Nyquist plots of as-assembled Na|NASICON|Na symmetric cell based on Carb-NZSP and NZSP.

**Figure S13:** Constant current cycling results for symmetric Na|SSE|Na cells. (a-b) 0.1 mA cm^-2^ and 0.1 mAh cm^-2^ for Carb-NZSP and NZSP, respectively. (c-d) 0.5 mA cm^-2^ and 0.5 mAh cm^-2^ cycling. (e) Cycling at 1.0 mA cm^-2^ and 1.0 mAh cm^-2^ cycling with NZSP short circuiting at 8.15 hours.


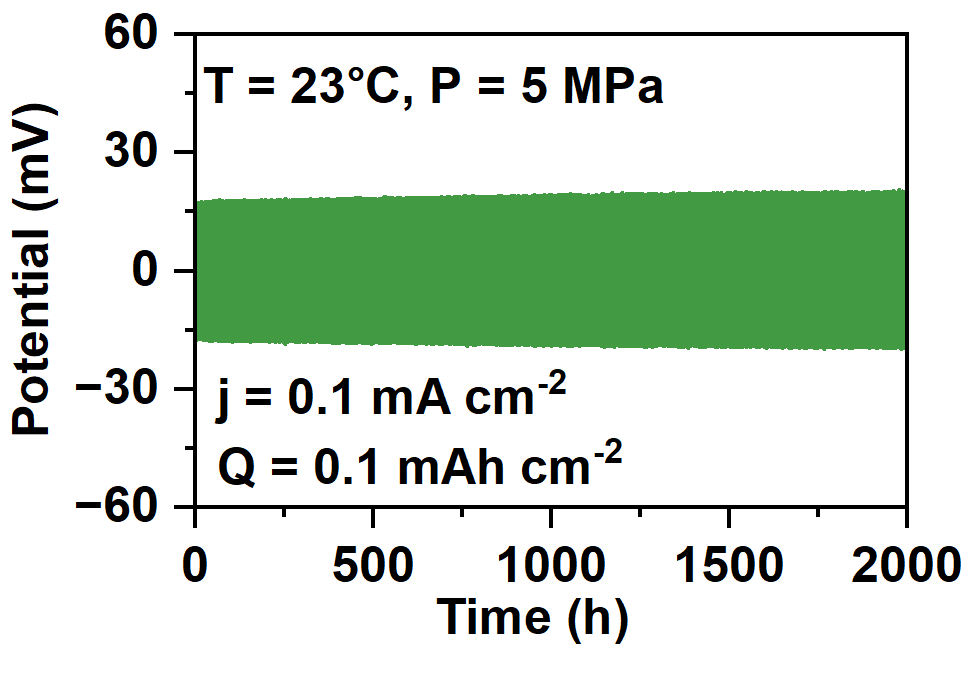

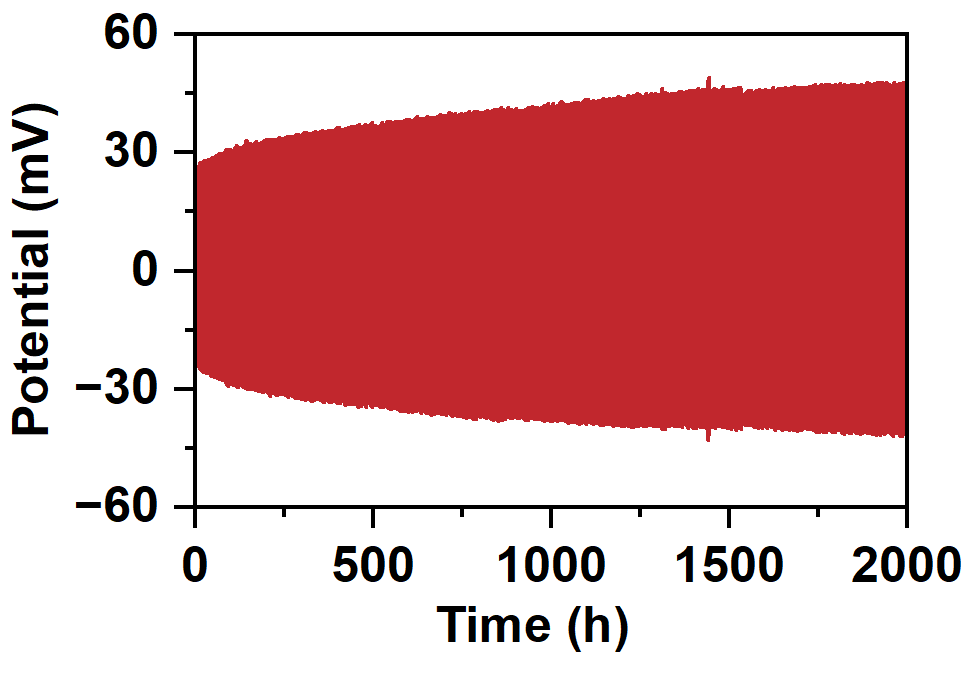

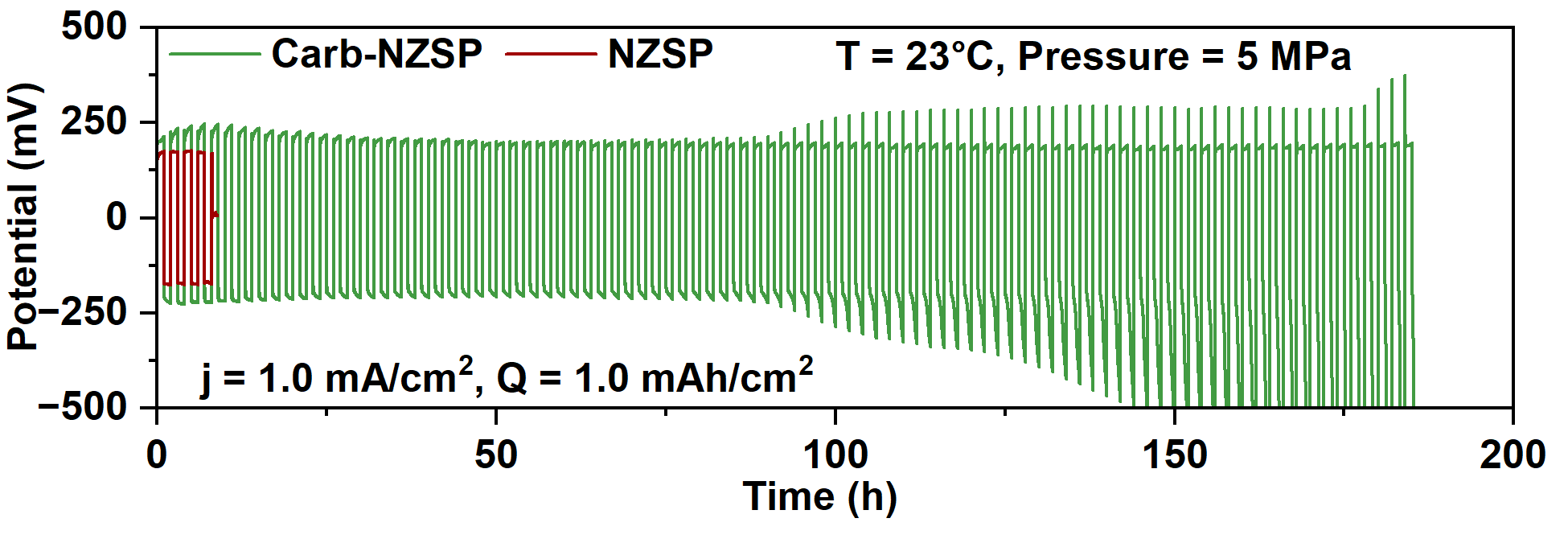

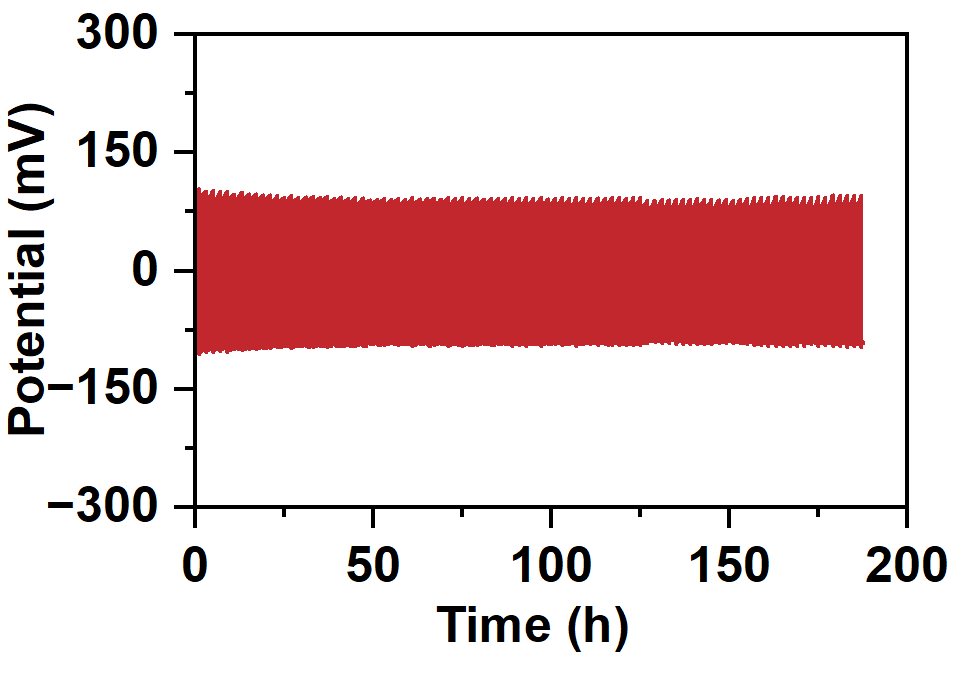

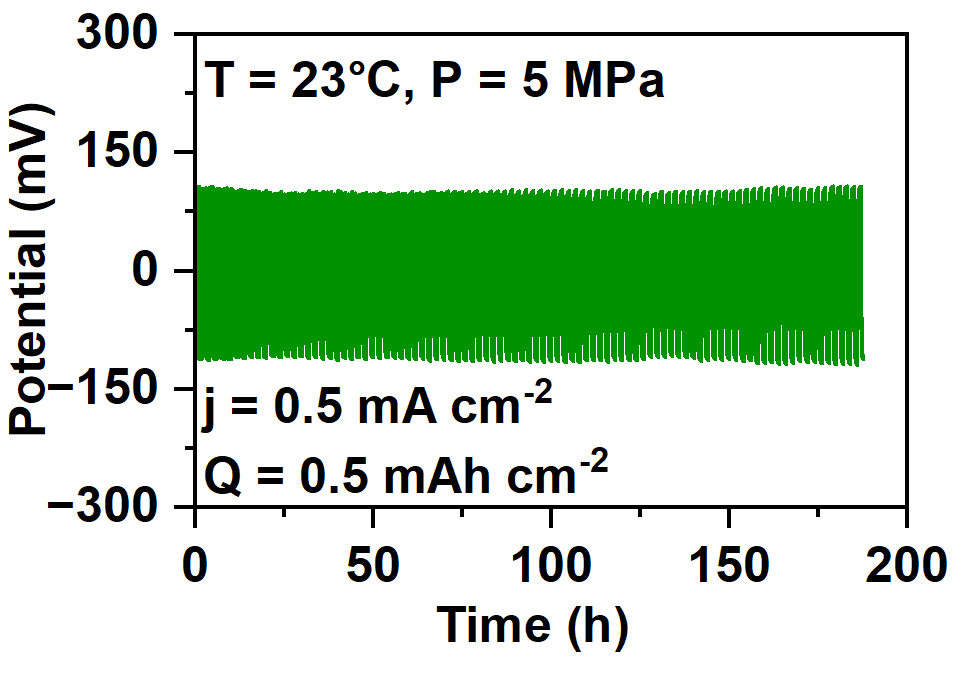


**a)**

**b)**

**c)**

**d)**

**e)**

**Figure S14:** Additional ccritical current density test with constant capacity of 0.1 mAh/cm^2^ at room temperature (a-b) for Carb-NZSP and (c-d) NZSP


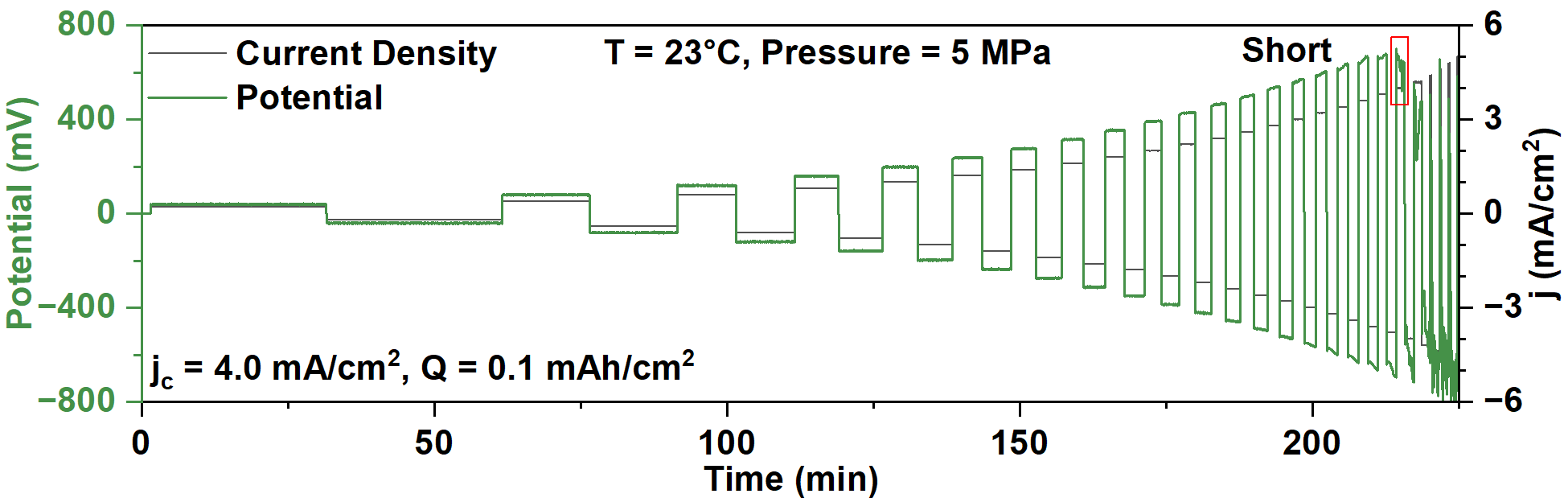

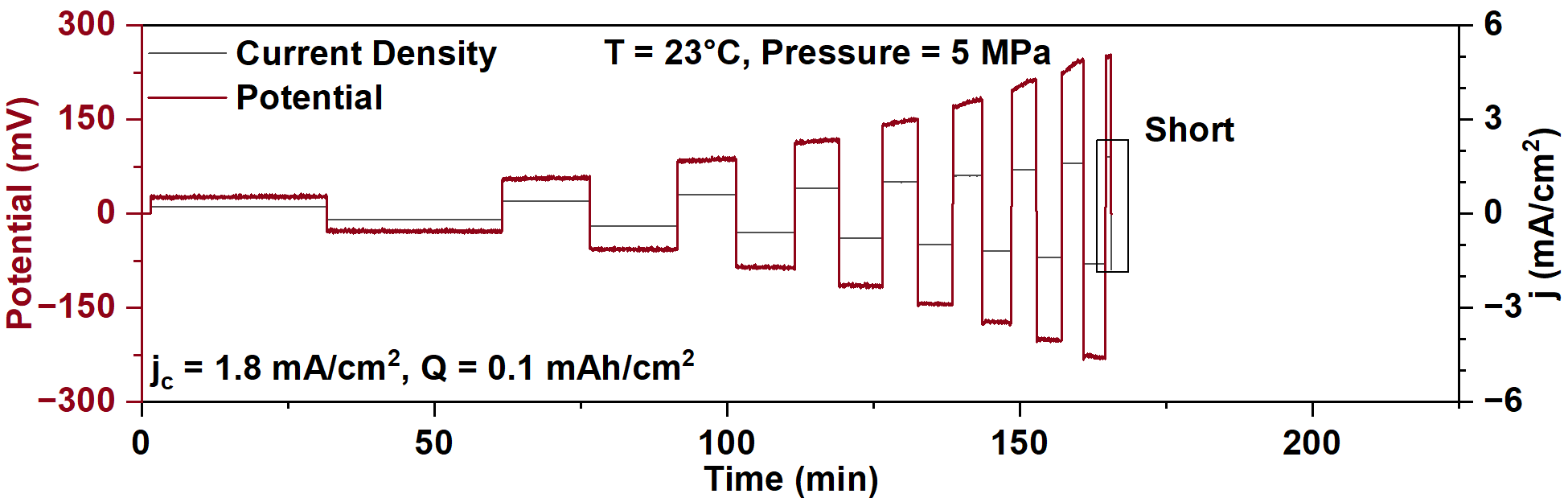

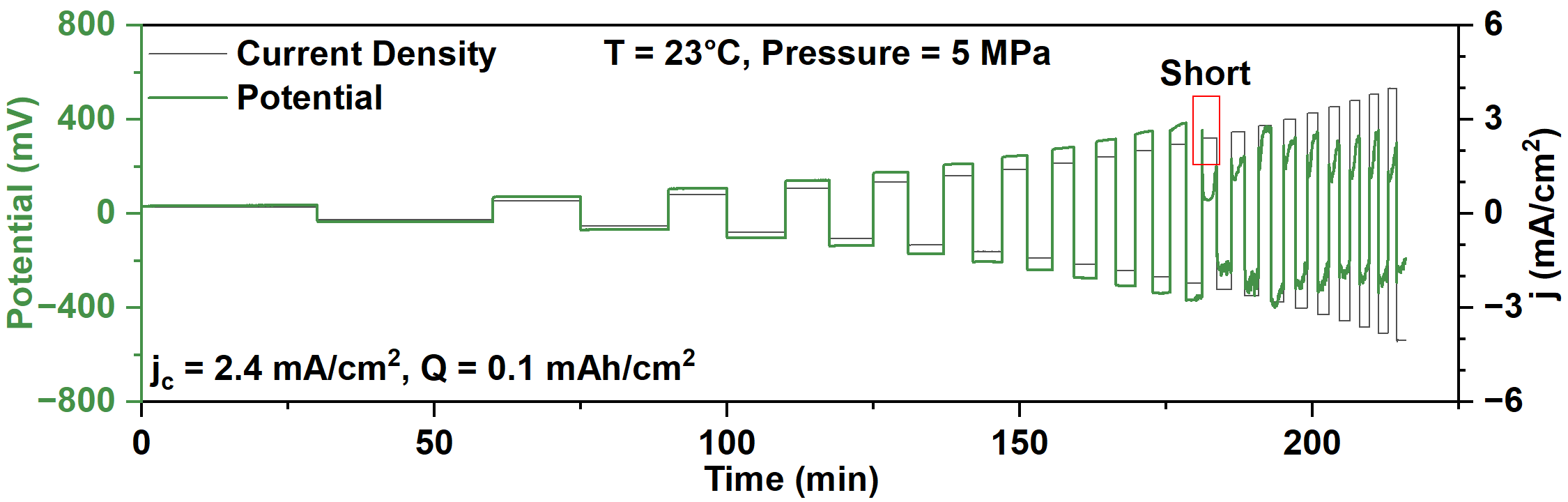

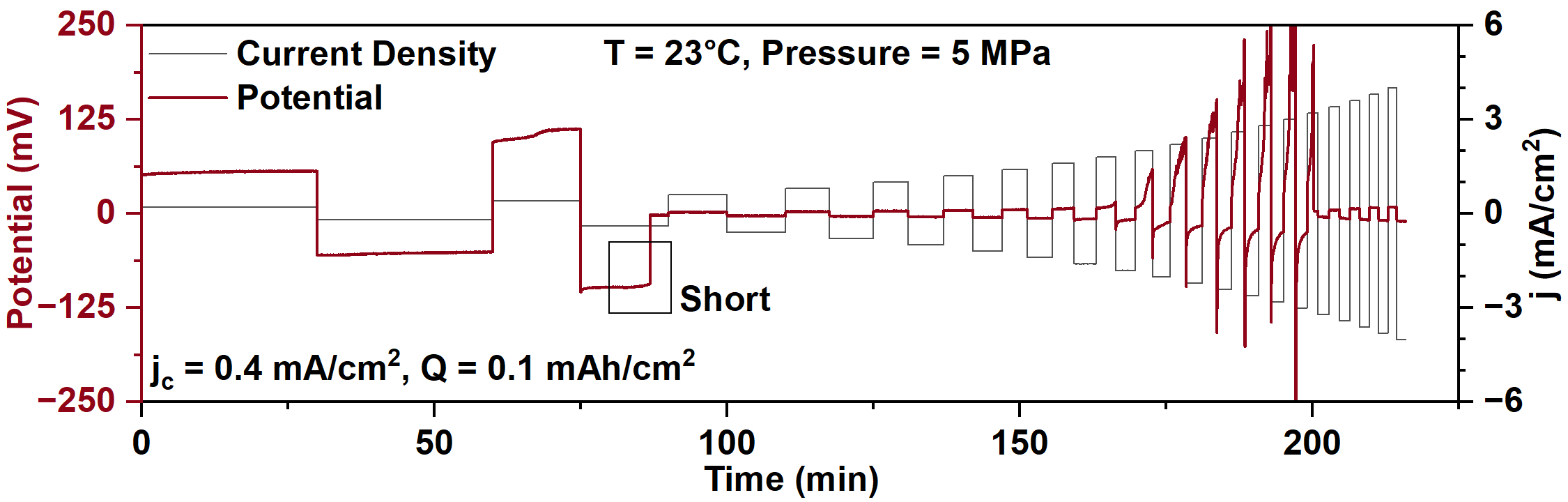


**a)**

**b)**

**c)**

**d)**

**Table S7.** Measurements of ionic conductivity at 23 °C on W|NZSP|W cells.

| NZSP Sample | 1 | | 2 | | 3 | | 4 | | 5 | |
| --- | --- | --- | --- | --- | --- | --- | --- | --- | --- | --- |
| χ^2^ | 6.86E-05 | | 1.02E-04 | | 9.32E-05 | | 1.30E-04 | | 7.80E-05 | |
| ^a)^ | Value | Error (%) | Value | Error (%) | Value | Error (%) | Value | Error (%) | Value | Error (%) |
| R_1_ | 57.4 | 1.2 | 75.1 | 1.5 | 68.4 | 1.4 | 70.5 | 1.5 | 88.2 | 1.3 |
| R_2_ | 138.0 | 0.9 | 136.0 | 2.4 | 131.0 | 1.6 | 124.0 | 2.2 | 139.0 | 2.5 |
| Q_1_-Yo | 7.2E-09 | 10.3 | 1.1E-08 | 19.2 | 9.6E-09 | 16 | 7.2E-09 | 19.2 | 8.5E-09 | 19 |
| Q_1_-n | 0.9 | 0.8 | 0.9 | 1.5 | 0.9 | 1.2 | 0.9 | 1.5 | 0.9 | 1.5 |
| R_3_ | 17200 | 16.6 | 699.0 | 16.2 | 1070 | 8.9 | 1140.0 | 45.3 | 175 | 31 |
| Q_2_-Yo | 2.6E-06 | 13.4 | 2.66E-06 | 22.7 | 7.2E-07 | 11.9 | 6.49E-06 | 25.2 | 8E-06 | 64 |
| Q_2_-n | 0.8 | 0.4 | 0.7 | 3.1 | 0.8 | 1.5 | 0.7 | 3.2 | 0.7 | 8.8 |
| Q_3_-Yo | 1.3E-06 | 10.7 | 4.99E-08 | 1.2 | 5.7E-08 | 1.2 | 7.11E-08 | 2.1 | 6.07E-08 | 0.9 |
| Q_3_-n | 0.9 | 4.4 | 1.0 | 0.2 | 1.0 | 0.2 | 1.0 | 0.4 | 1.0 | 0.2 |

| Carb-NZSP | 1^b)^ | | 2 | | 3 | |
| --- | --- | --- | --- | --- | --- | --- |
| χ^2^ | 2.51E-04 | | 2.63E-04 | | 1.08E-04 | |
| ^a)^ | Value | Error (%) | Value | Error (%) | Value | Error (%) |
| R_1_ | 72.3 | 2.2 | 68.9 | 1.7 | 71.4 | 1.1 |
| R_2_ | 4860 | 31 | 104.0 | 1.8 | 2750 | 200 |
| Q_1_-Yo | 2.54E-06 | 8.3 | 7.4E-09 | 22.1 | 1.1E-05 | 43.6 |
| Q_1_-n | 0.7 | 4.9 | 0.9 | 1.7 | 0.7 | 19.9 |
| R_3_ | 102.0 | 3.3 | 3760 | 80.6 | 125.0 | 1.0 |
| Q_2_-Yo | 5.5E-09 | 30 | 4.2E-06 | 6.2 | 7.7E-09 | 12.5 |
| Q_2_-n | 0.9 | 2.3 | 0.8 | 9.3 | 0.9 | 1.0 |
| Q_3_-Yo | 6.1E-06 | 16.1 | 3.5E-06 | 26.4 | 5.35E-06 | 35.2 |
| Q_3_-n | 0.7 | 8.9 | 0.8 | 12.7 | 0.8 | 21.8 |

^a)^ R in Ω, Q-Y_o_ in F

^b)^ RL(RQ)(RQ)Q circuit used for cell #1 to account for extended cable used during activation energy measurement. L = 104 H ± 1.8%

**Table S8.** Measurements of ionic conductivity at 23 °C on W|Carb-NZSP|W cells.

^a)^ R in Ω, Q-Y_o_ in F

**
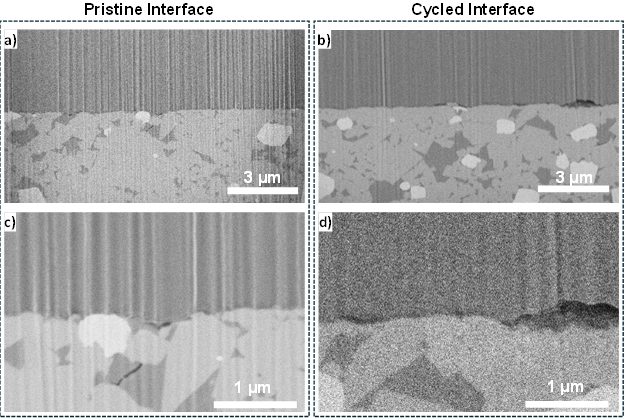
**

**Figure S15.** Cryo-SEM-FIB backscatter images of the interface between sodium metal and baseline NZSP are shown in the a) pristine and b) post-mortem states. High magnification images show (c) good adhesion between Na metal and NZSP in the pristine state, with little to no delamination and some glass cracking, while (d) the cycled interface shows more significant porosity at the interface.

**Figure S16.** Electrochemical profiles for cells analyzed for dendrites in cryo-FIB. (a) Galvanostatic data for Ni|Na|NZSP|Na|Ni symmetric cell at 0.5 mA cm^-2^ with a five-cycle activation at 0.1 mA cm^-2^ with 0.1 mAh cm^-2^ capacity. Shorting is observed during the first half cycle at 0.5 mA cm^-2^. b) Ni|Te|NZSP|Na|Ni Asymmetric cell at a current density of 0.1 mA cm^-2^ and a constant capacity of 0.1 mAh cm^-2^ with a 1V upper voltage cutoff, shorting observed on the third cycle. c) magnified view of (b). d) Asymmetric cell configuration with Carb-NZSP, shorting on the 9^th^ cycle at 1 mA cm^-2^.


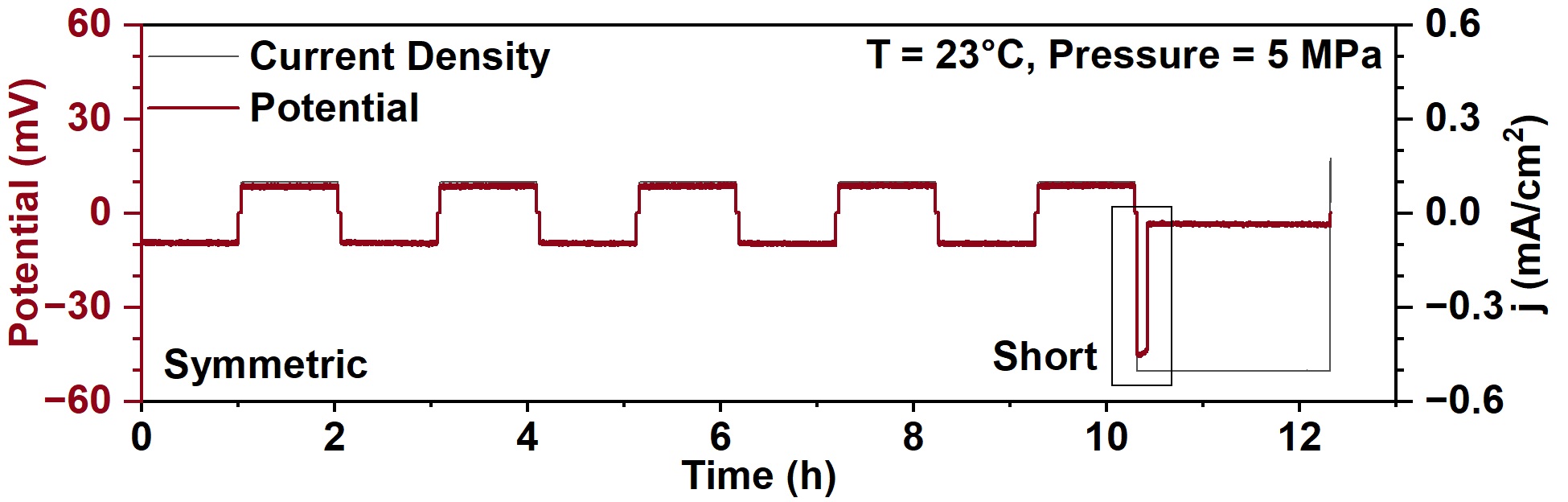


**a)**

**b)**


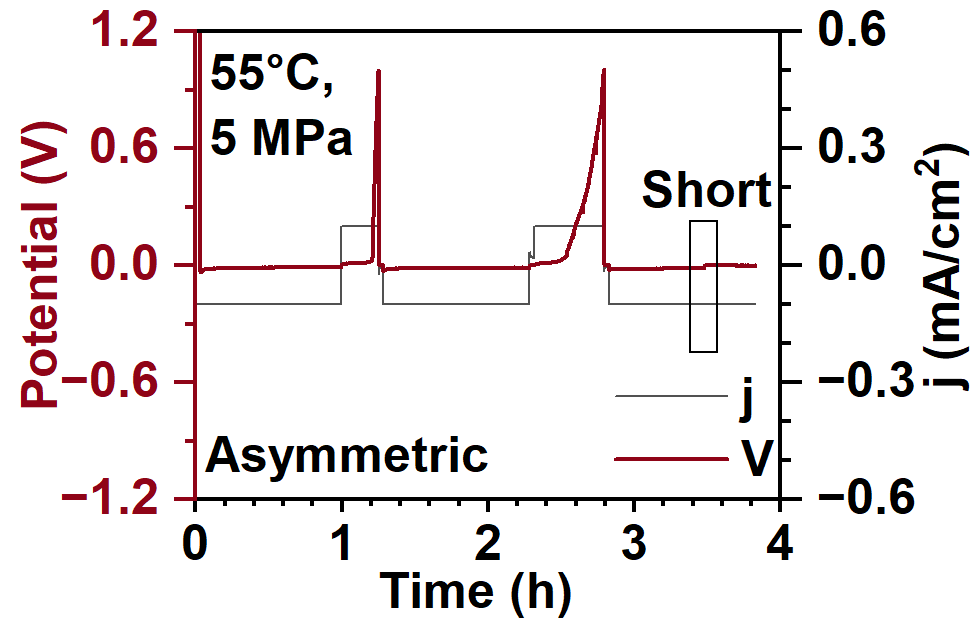

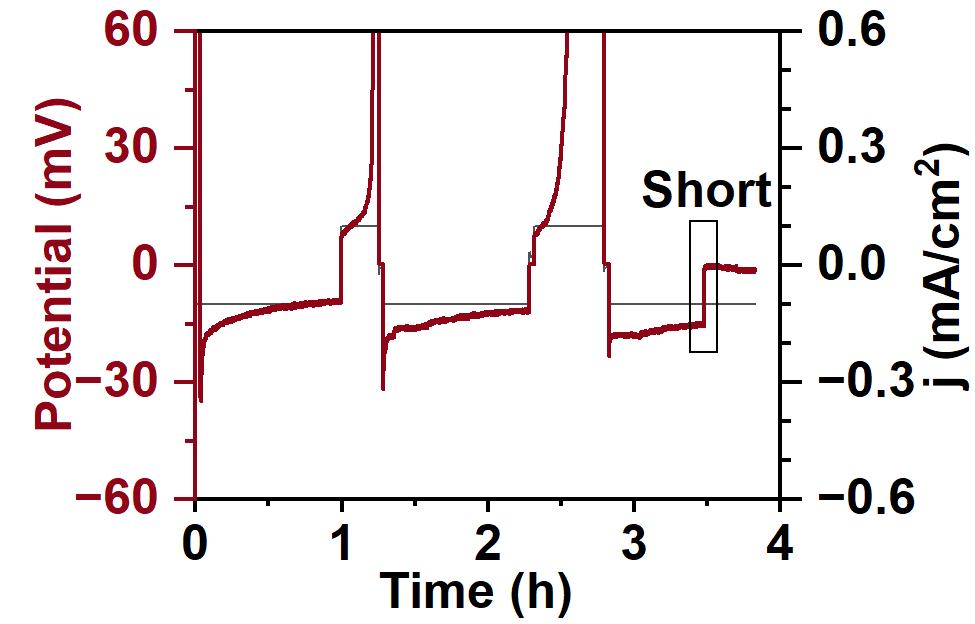


**c)**


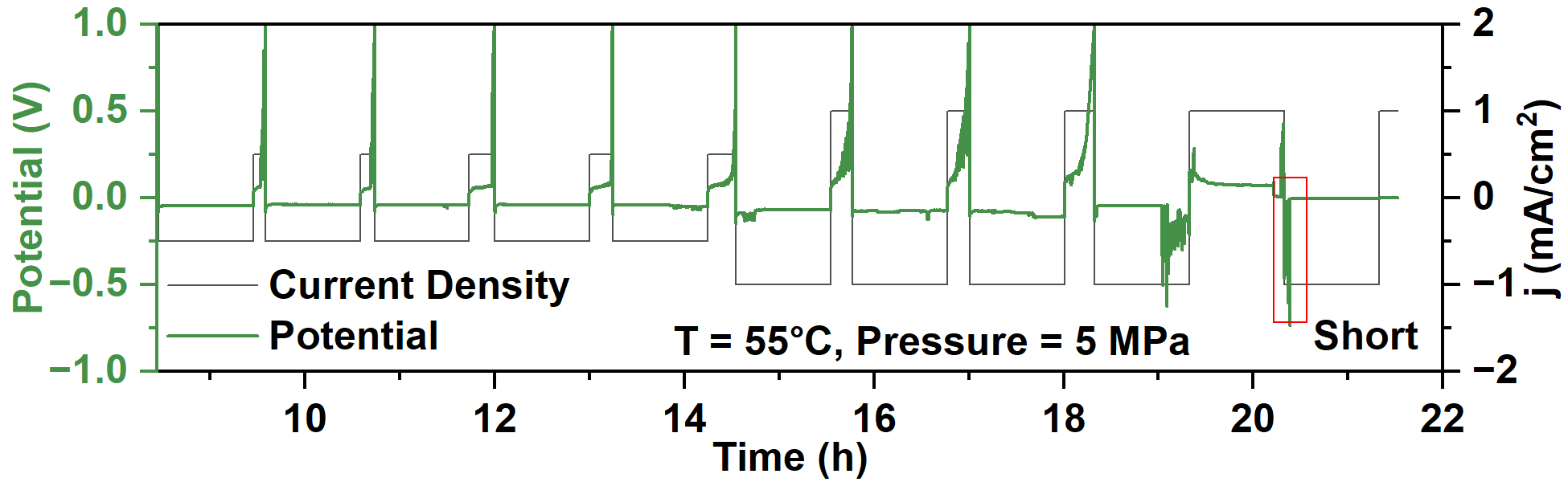


**d)**


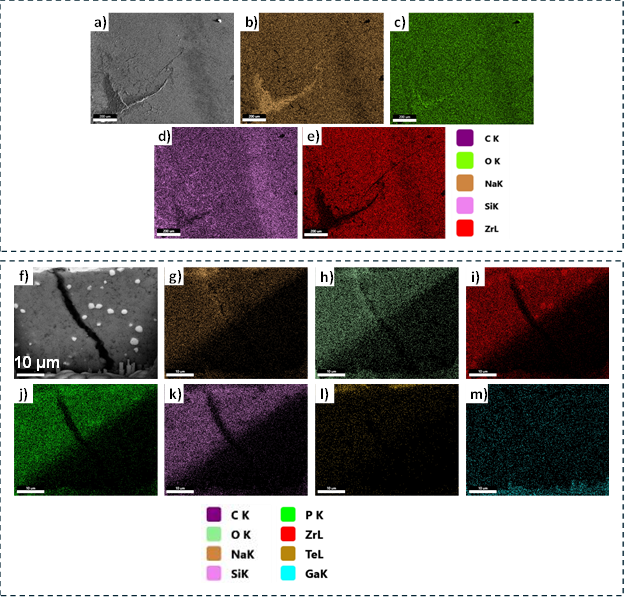


**Figure S17:** (a-e) Secondary electron image of the sodium metal dendrite found on the surface of a Na|NZSP|Na symmetric cell, and all corresponding EDX Maps. (f-m) BSE image of the sodium metal dendrite found on the surface of a Na|NZSP|Te|Ni asymmetric cell, and all corresponding EDX Maps.

| Method/  Additive^a)^ | ρ  [%] | Conductivity  [mS cm^-1^] | E_a_  [eV] | Temp  [°C] | CCD  [mA  cm^-2^] | Capacity  [mAh  cm^-2^] | Max Cycle  duration  [hr @  mA cm^-2^] | P  [MPa] | Ref. |
| --- | --- | --- | --- | --- | --- | --- | --- | --- | --- |
| **Carb-NZSP** | **93** | **1.08** | **0.34** | **23** | **2.4** | **0.1** | **1000 @ 0.1** | **5** | **This**  **Work** |
| Liq. Phase | 93 | 0.60 | 0.286 | RT | -- | -- | -- | -- | ^[8]^ |
| SS | 87 | 0.61 | -- | 25 | <0.1 | 0.008^b)^ | 12 @ 0.1 | -- | ^[9]^ |
| SS | -- | 0.76 | 0.30 | RT | 0.2 | 0.2^b)^ | 300 @ 0.1 | coin | ^[10]^ |
| SS + Na | -- | 0.4 | -- | RT | 0.15 | 0.15 | 95 @ 0.1 | coin | ^[11]^ |
| SS + Na/P | -- | 0.23 | 0.47 | 22 | 0.2 | 0.1 | 70 @ 0.1  60°C | coin | ^[12]^ |
| UHS + Na/P | -- | 0.28 | 0.36 | RT | 0.6 | 0.3^b)^ | 4800 @ 0.1 | -- | ^[13]^ |
| NZ**Lu**_0.3_SP | _--_ | 0.77 | 0.30 | RT | 1.4 | 0.7^b)^ | 4800 @ 0.1 | -- | ^[13]^ |
| SS | -- | 0.41 | 0.344 | 25 | 0.15 | 0.15^b)^ | 325 @ 0.05 | coin | ^[14]^ |
| SS | ~96 | 0.4 | 0.36 | RT | -- | -- | -- | -- | ^[15]^ |
| SG 2.4 | 95-99 | -- | -- | RT | 14 | 0.5 | 1000 @1  180 @ 3  13 @ 5 | 6-12 | ^[16]^ |
| SS | 91 | 1.13 | 0.32 | 20 | -- | -- | -- | -- | ^[17]^ |
| Spark Plasma | ~100 | 1.8 | -- | 25 | -- | -- | -- | -- | ^[18]^ |
| Liq Phase Na_3_BO_3_ | 93 | 1.4 | 0.29^c)^ | 25 | -- | -- | -- | -- | ^[19]^ |

^a)^ SS = ‘solid state synthesis’, SG x = Sol-Gel synthesis and x in Na_1+x_Zr_2_Si_x_P_3-x_O_12_, “+ Na/P” = reported excess of Na or P in synthesis.

^b)^ Constant time CCD test listed as maximum capacity in one cycle.

^c)^ Converted from kJ mol^-1^

**Table S9:** Total ionic conductivity, activation energy, critical current density, and cycling duration in literature values.

**Table S10:** Calculated values of enthalpy of formation from Materials Project Database.

| Formula | Calculated  (eV/f.u.) | Calculated  (kJ mol⁻¹) | Carb-NZSP  (mol/rxn) | Carb-NZSP  (kJ/rxn) | NZSP  (mol/rxn) | NZSP  (kJ/rxn) |
| --- | --- | --- | --- | --- | --- | --- |
| Na₂CO₃ | -12.5 | -1201.9 | 1.5 | 1802.8 | 1.5 | 1802.8 |
| SiO₂ | -9.8 | -947.3 | 0.0 | 0.0 | 2.0 | 1894.7 |
| ZrO₂ | -11.4 | -1103.9 | 0.0 | 0.0 | 2.0 | 2207.8 |
| PH₆NO₄ | -17.7 | -1708.0 | 1.0 | 1708.0 | 1.0 | 1708.0 |
| SiC | -0.6 | -59.0 | 2.0 | 118.1 | 0.0 | 0.0 |
| ZrC | -2.0 | -195.6 | 2.0 | 391.2 | 0.0 | 0.0 |
| Na₃Zr₂Si₂PO₁₂ | -63.0 | -6076.0 | 1.0 | -6076.0 | 1.0 | -6076.0 |
| CO₂ | -5.3 | -512.2 | 5.5 | -2817.1 | 1.5 | -768.3 |
|  | -3.8 | -371.0 | 1.5 | -556.5 | 1.5 | -556.5 |
| H₃N | -1.7 | -163.1 | 1.0 | -163.1 | 1.0 | -163.1 |
|  |  |  |  | -5592.6 |  | 49.3 |

**Figure S18.** Schematic method of image thresholding for particle analysis used to quantify each phase. Thresholding of the porosity/glassy regions shown here for example, separated manually after particle analysis, and qualitatively referenced to original images.

Standard Automatic Thresholding

Removing shadowing

*Manually separate pores vs glassy regions*


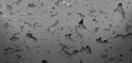

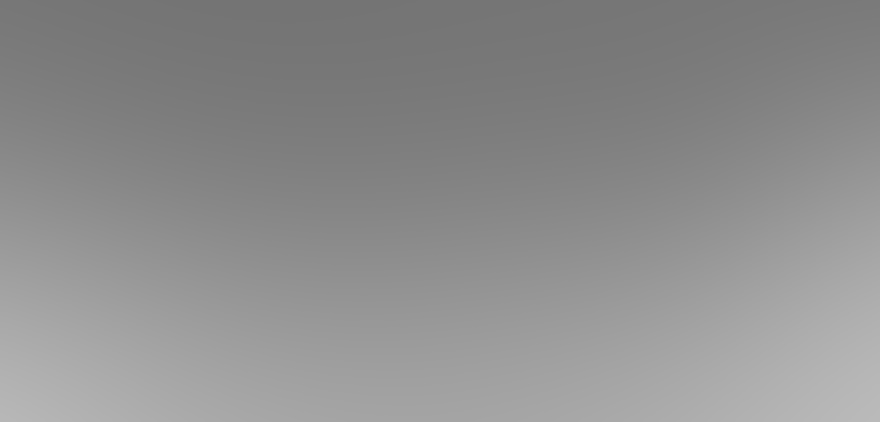

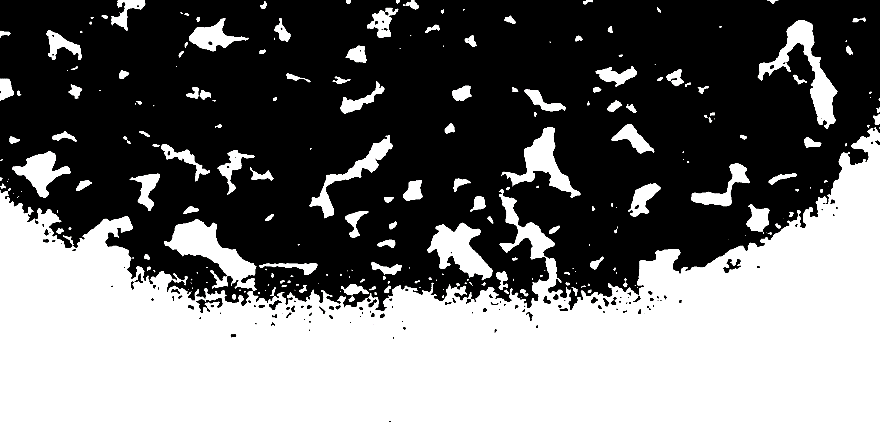

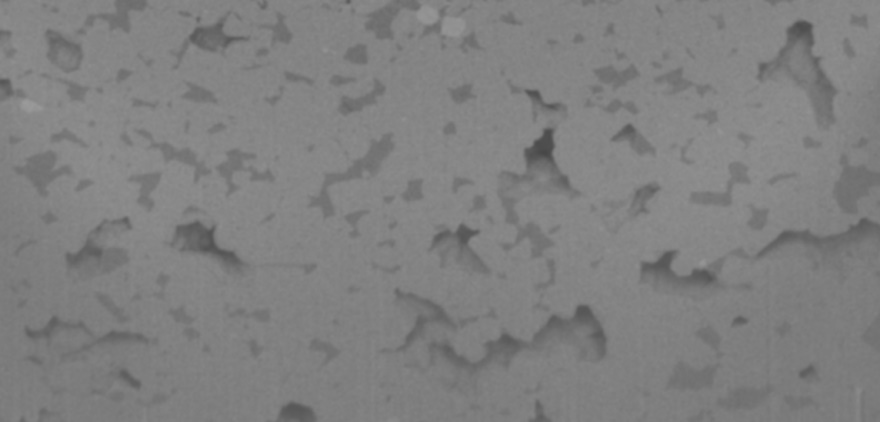

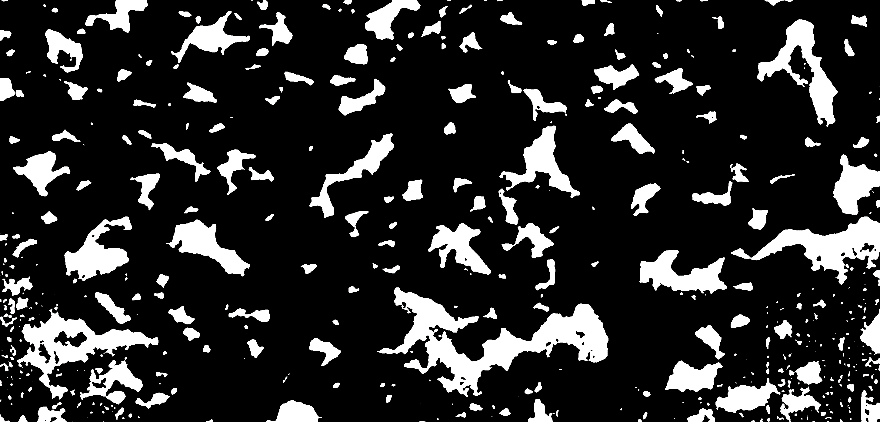


Average

Gaussian Blur

/ Invert


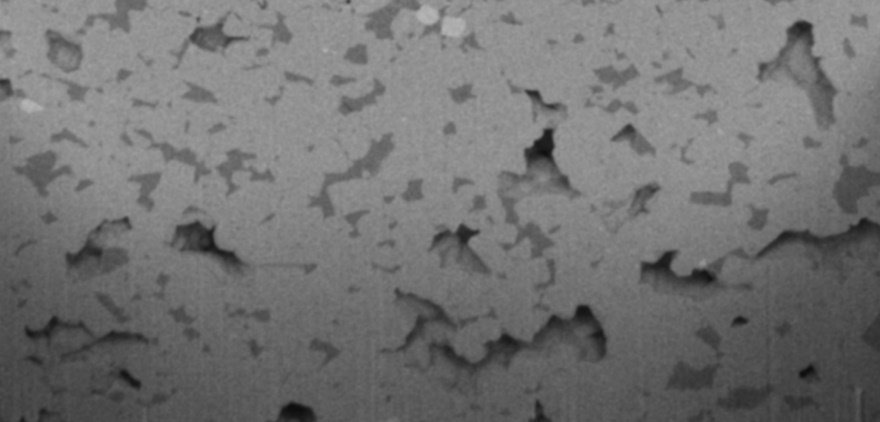

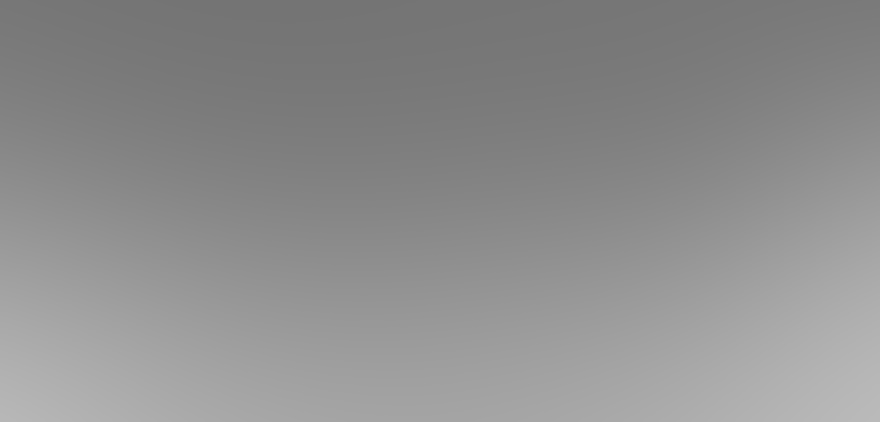

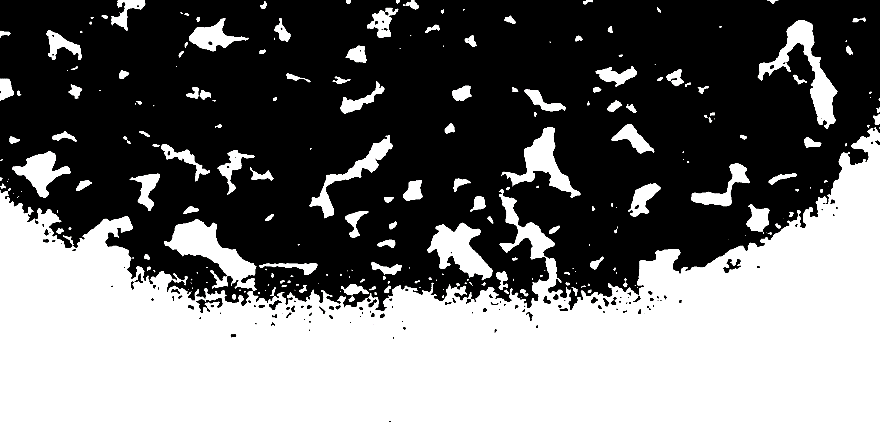

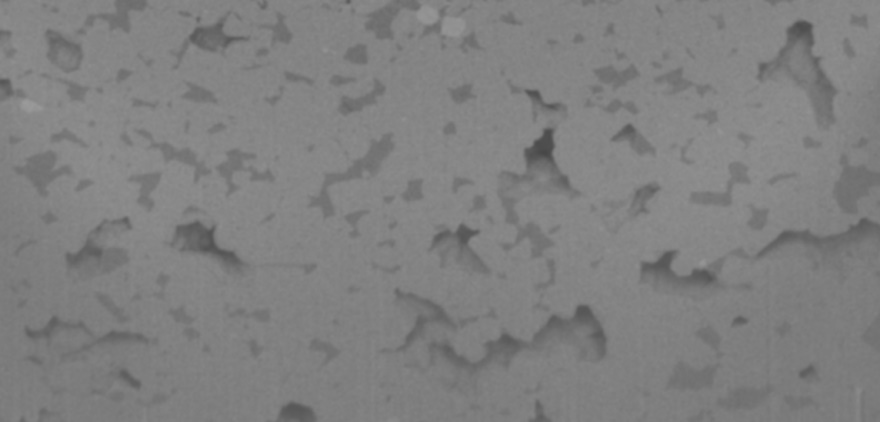

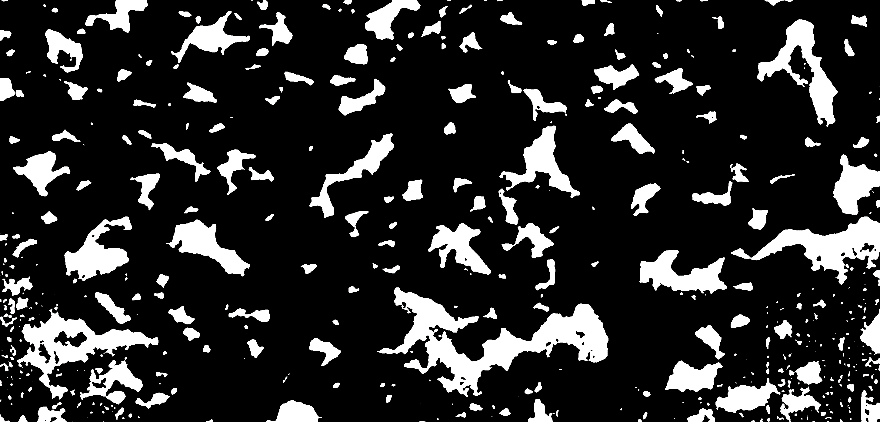


Original Image (cropped)

**Figure S19:** Elevated temperature cycling results for Na|Carb-NZSP|Na cells. (a) 0.5 mA cm^-2^ and 0.5 mAh cm^-2^ at 55°C following 5 formation cycles at 0.1 mA cm^-2^ for 0.1 mAh cm^-2^. (b) 0.5 mA cm^-2^ and 0.5 mAh cm^-2^ cycling, followed by 3.0 mA cm^-2^ and 3.0 mAh cm^-2^ with polarization observed around 15.8 hours. c) Full cell NVP|LE|NZSP|Na select cycle voltage profiles for C/10 cycling. d) Full cell C/10 specific capacities and Coulombic efficiency (CE).


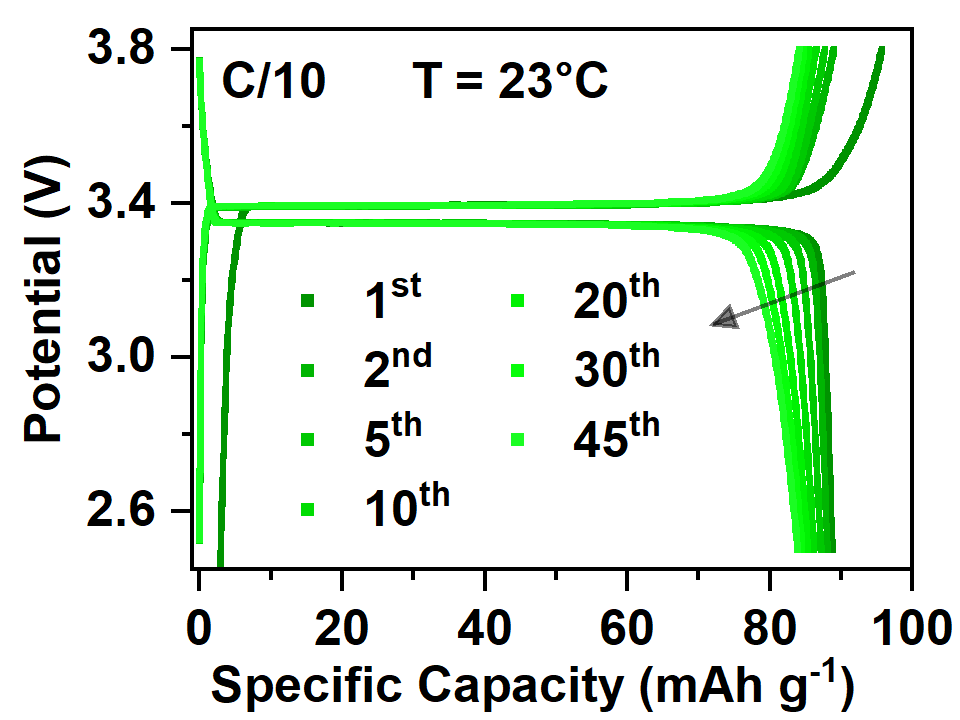

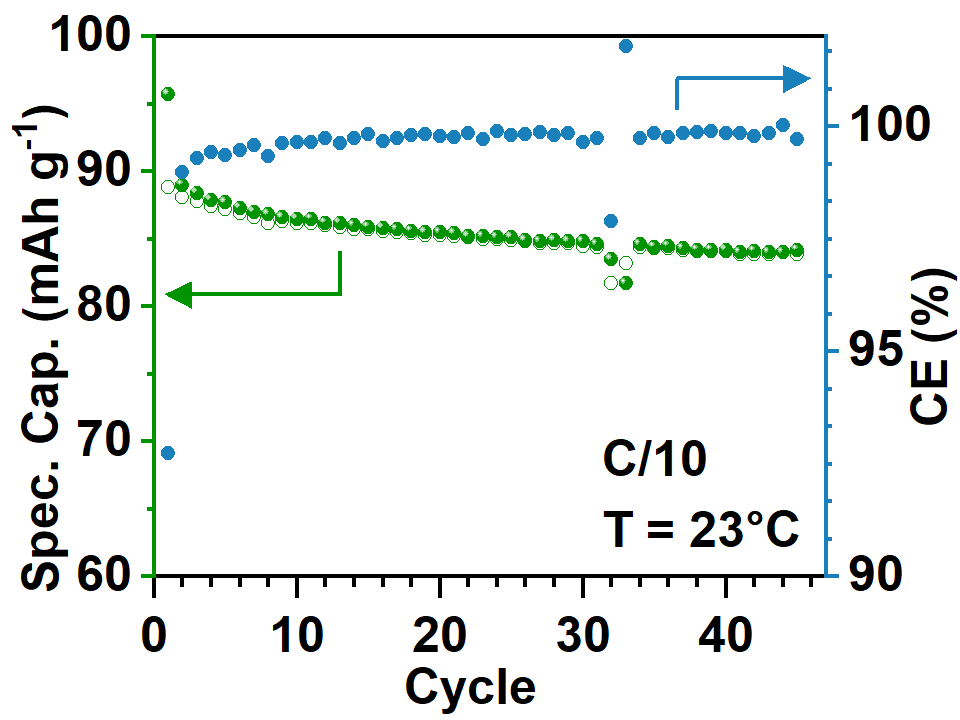

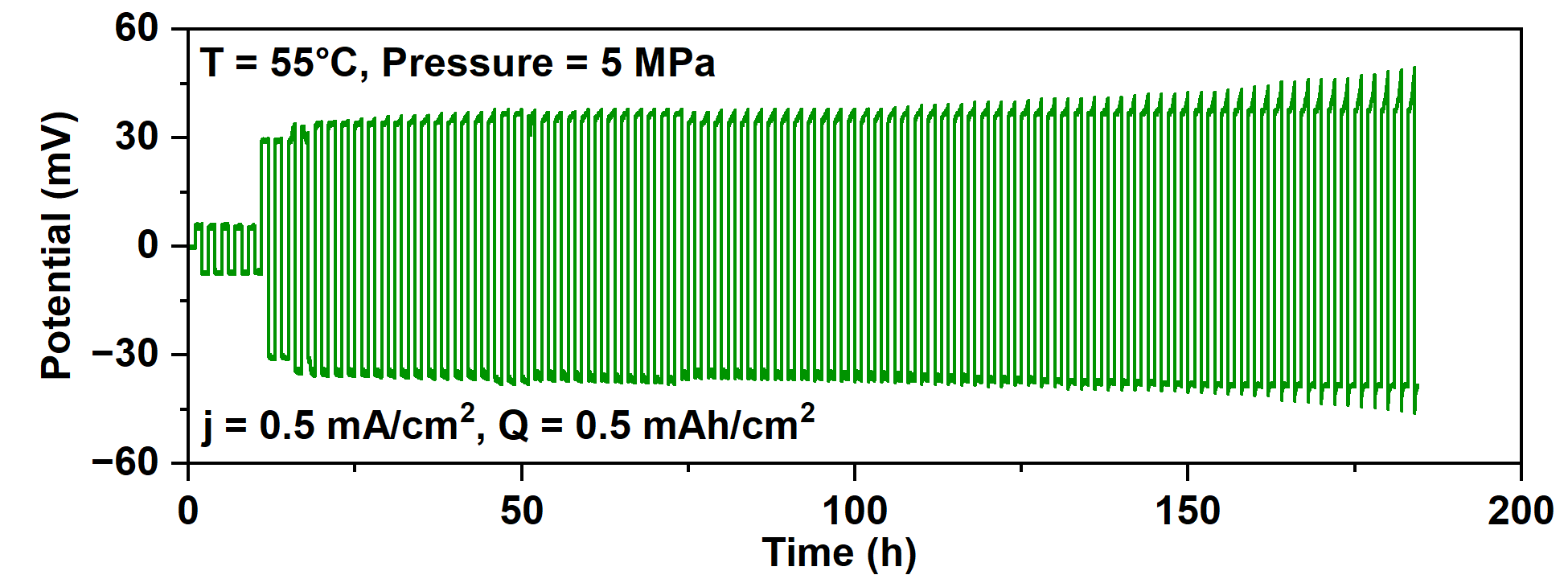

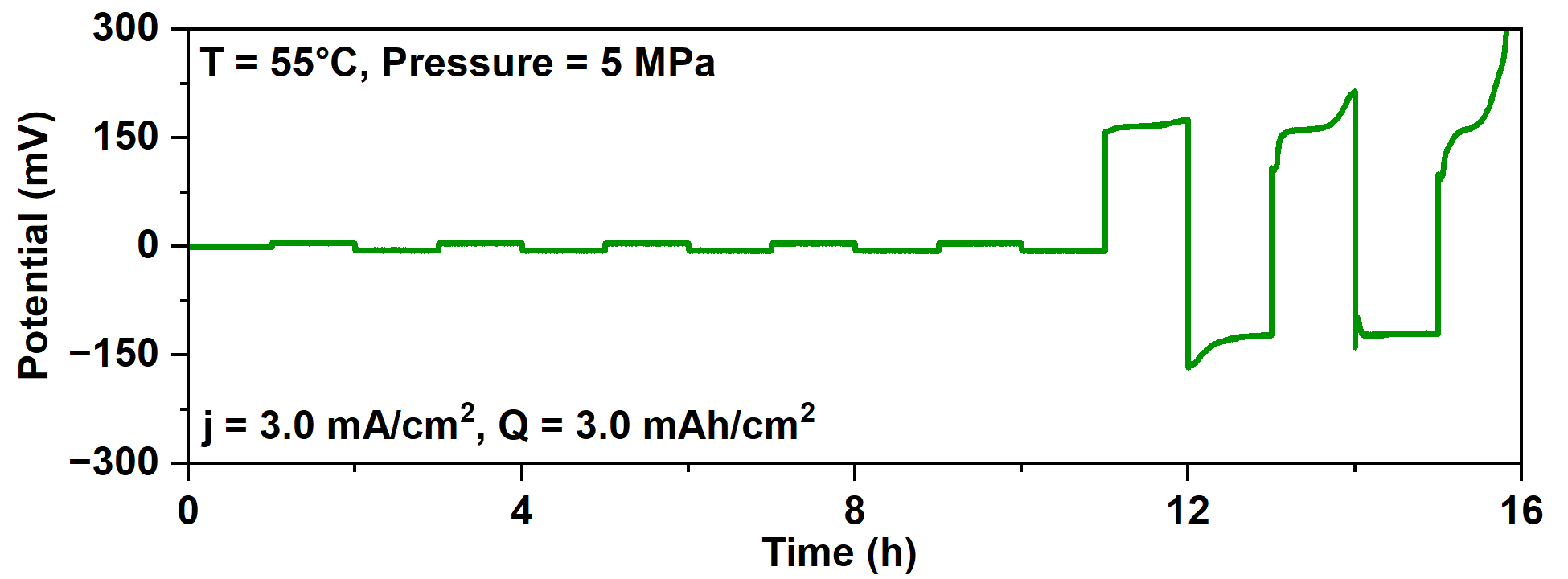


**a)**

**b)**

**c)**

**d)**

**References**

[1] E. Quérel, I. D. Seymour, A. Cavallaro, Q. Ma, F. Tietz, A. Aguadero, *JPhys Energy* **2021**, *3*, DOI 10.1088/2515-7655/ac2fb3.

[2] J. C. Russ, R. T. Dehoff, *Practical Stereology*, Springer US, Boston, MA, **2000**.

[3] S. Monismith, C. D. Fincher, Y. M. Chiang, J. Qu, R. Dingreville, *Adv Energy Mater* **2024**, *14*, DOI 10.1002/aenm.202303567.

[4] M. Ambati, T. Gerasimov, L. De Lorenzis, *Comput Mech* **2015**, *55*, 383.

[5] C. Miehe, F. Welschinger, M. Hofacker, *Int J Numer Methods Eng* **2010**, *83*, 1273.

[6] J. A. Stewart, R. Dingreville, *Acta Mater* **2020**, *188*, 181.

[7] R. Dingreville, J. Stewart, E. Chen, J. Monti, *Benchmark Problems for the Mesoscale Multiphysics Phase Field Simulator (MEMPHIS)*, Albuquerque, NM, and Livermore, CA (United States), **2020**.

[8] J. A. S. Oh, L. He, A. Plewa, M. Morita, Y. Zhao, T. Sakamoto, X. Song, W. Zhai, K. Zeng, L. Lu, *ACS Appl Mater Interfaces* **2019**, *11*, 40125.

[9] Z. Gao, J. Yang, G. Li, T. Ferber, J. Feng, Y. Li, H. Fu, W. Jaegermann, C. W. Monroe, Y. Huang, *Adv Energy Mater* **2022**, *12*, DOI 10.1002/aenm.202103607.

[10] Y. Zhao, C. Wang, Y. Dai, H. Jin, *Nano Energy* **2021**, *88*, 106293.

[11] Z. Sun, L. Li, C. Sun, Q. Ni, Y. Zhao, H. Wu, H. Jin, *Nano Lett* **2022**, DOI 10.1021/acs.nanolett.2c02509.

[12] X. Miao, H. Di, X. Ge, D. Zhao, P. Wang, R. Wang, C. Wang, L. Yin, *Energy Storage Mater* **2020**, *30*, 170.

[13] D. Zuo, L. Yang, Z. Zou, S. Li, Y. Feng, S. J. Harris, S. Shi, J. Wan, *Adv Energy Mater* **2023**, *13*, DOI 10.1002/aenm.202301540.

[14] D. Li, C. Sun, C. Wang, J. Li, Z. Wang, H. Jin, *Energy Storage Mater* **2023**, *54*, 403.

[15] R. O. Fuentes, F. M. Figueiredo, F. M. B. Marques, J. I. Franco, *J Eur Ceram Soc* **2001**, *21*, 737.

[16] Q. Ma, T. Ortmann, A. Yang, D. Sebold, S. Burkhardt, M. Rohnke, F. Tietz, D. Fattakhova-Rohlfing, J. Janek, O. Guillon, *Adv Energy Mater* **2022**, *12*, DOI 10.1002/aenm.202201680.

[17] S. Narayanan, S. Reid, S. Butler, V. Thangadurai, *Solid State Ion* **2019**, *331*, 22.

[18] J. S. Lee, C. M. Chang, Y. Il Lee, J. H. Lee, S. H. Hong, *Journal of the American Ceramic Society* **2004**, *87*, 305.

[19] K. Noi, K. Suzuki, N. Tanibata, A. Hayashi, M. Tatsumisago, *Journal of the American Ceramic Society* **2018**, *101*, 1255.
